# An autoinhibitory mechanism modulates MAVS activity in antiviral innate immune response

**DOI:** 10.1038/ncomms8811

**Published:** 2015-07-17

**Authors:** Yuheng Shi, Bofeng Yuan, Nan Qi, Wenting Zhu, Jingru Su, Xiaoyan Li, Peipei Qi, Dan Zhang, Fajian Hou

**Affiliations:** 1 State Key Laboratory of Cell Biology, Innovation Center for Cell Signaling Network, Institute of Biochemistry and Cell Biology, Shanghai Institutes for Biological Sciences, Chinese Academy of Sciences, Shanghai 200031, China

## Abstract

In response to virus infection, RIG-I senses viral RNA and activates the adaptor protein MAVS, which then forms prion-like filaments and stimulates a specific signalling pathway leading to type I interferon production to restrict virus proliferation. However, the mechanisms by which MAVS activity is regulated remain elusive. Here we identify distinct regions of MAVS responsible for activation of transcription factors interferon regulatory factor 3 (IRF3) and nuclear factor kappa-light-chain-enhancer of activated B cells (NF-κB). These IRF3- and NF-κB-stimulating regions recruit preferential TNF receptor-associated factors (TRAFs) for downstream signalling. Strikingly, these regions' activities are inhibited by their respective adjacent regions in quiescent MAVS. Our data thus show that an autoinhibitory mechanism modulates MAVS activity in unstimulated cells and, on viral infection, individual regions of MAVS are released following MAVS filament formation to activate antiviral signalling cascades.

## Abstract (web summary)

The cellular protein RIG-I detects viral RNA and activates another protein, MAVS, which then forms filaments and stimulates an antiviral pathway. Here, the authors identify different regions within MAVS involved in activating transcription factors IRF3 and NF-κB, and in MAVS self-inhibition.

## Graphical abstract

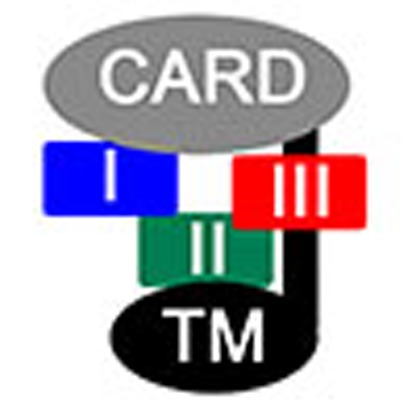

As a response to virus infection, an evolutionarily conserved innate immune mechanism is executed to induce type I interferon (IFN) and proinflammatory cytokines in higher organisms^1^,^2^. Antiviral innate immune response is initiated with the detection of viral nucleic acids by pattern recognition receptors in host cells. There are two types of pattern recognition receptor, membrane-bound and cytosolic ones, to sense viral RNA featuring double-strand and 5'-phosphate modification^3^,^4^. Toll-like receptor 3, localized on the endosomal membrane, recognizes extracellular double-stranded RNA^3^,^5^. Its cytosolic counterpart is retinoic acid-inducible gene I (RIG-I)-like receptors (RLR), including RIG-I, MDA5 and LGP2. RIG-I and MDA5 sense cytosolic base-paired RNA in antiviral signalling, whereas LGP2 plays a modulatory role^6^,^7^,^8^,^9^,^10^.

RIG-I binds to base-paired RNA with its helicase and C-terminal regulatory domain, resulting in a conformational switch to release its N-terminal 2CARD domains^11^,^12^,^13^,^14^, which then activate MAVS with the help of K63-linked ubiquitin chains^15^,^16^. MAVS (also known as IPS-1, VISA and CARDIF), located mostly on mitochondrial outer membrane, plays a critical role in antiviral innate immune signalling^17^,^18^,^19^,^20^. A fraction of MAVS is also located to peroxisome for rapid response to virus infection^21^ and mitochondrial-associated membrane is reported to be a site for MAVS signalling^22^. On viral infection, RIG-I induces MAVS to form prion-like filaments through its N-terminal CARD domain^23^. Recent structural study revealed that the helical tetrameric structure formed by the RIG-I CARD domains acts as a template for the rigid-body assembly of the MAVS CARD domain to form filament^24^,^25^. MAVS filament is a potent activator for downstream signalling effectors, such as inhibitor of kappa B kinase (IKK) and TBK1, which is mediated by multiple TRAFs^26^. In addition, MAVS activity is modulated by many factors of host cells and viruses^27^. For example, WDR5, TOM70 and IFIT3 could facilitate MAVS signalling^28^,^29^,^30^. NLRX1 and MFN2 have been reported to inhibit MAVS-mediated type I interferon signalling through direct protein–protein interaction^31^,^32^. MAVS is also regulated via degradation mechanism by PCBP2-AIP4, Ndfip1-smurf1, COX5B-autohpagy, TRIM25 and Gp78 (refs 33, 34, 35, 36, 37).

Accumulating evidence indicates that MAVS becomes active by forming prion-like filaments in response to virus infection. Prion-like filament formation does not involve protein upregulation or post-translational modification, raising an important question on how MAVS acquires activity during the process. On the other hand, the mechanism that keeps MAVS inactive in the absence of stimulation is largely unknown.

In this study, we investigate three functionally distinct active regions of MAVS for the activation of its downstream effectors. We identify a region of MAVS comprising amino acids 401–450 (Region III) for TBK1/IRF3 activation. We also define two regions of MAVS (Region I aa-138–152 and Region II aa-451–465) responsible for IKK/NF-κB activation. Furthermore, we discover that Region II and Region III are inhibited by their respective adjacent regions in the absence of upstream signal and are activated with the formation of MAVS filaments on stimulation. Collectively, our data reveal an autoinhibitory mechanism that regulates these active regions in unstimulated MAVS, thus ensuring a prompt responsiveness of MAVS to virus infection.

## Results

### Identification of MAVS Region III for TBK1/IRF3 activation

**Figure 1:**
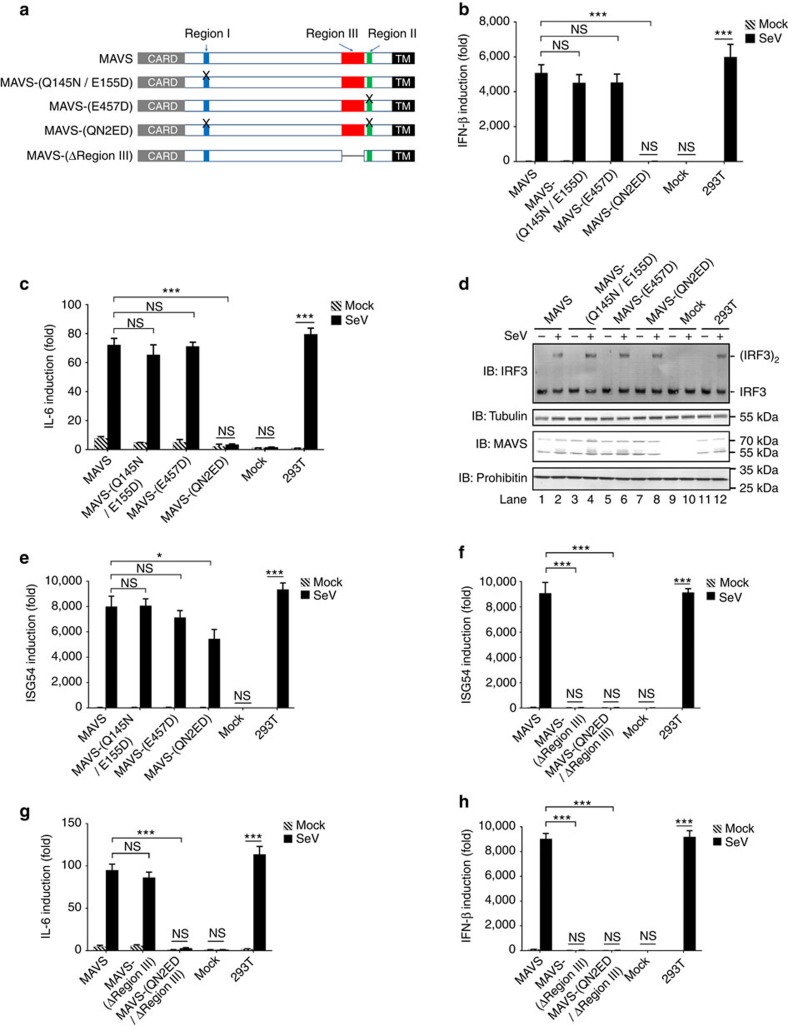
The putative TRAF-binding motifs in Region I and Region II are required for MAVS to activate IKK/NF-κB but not for its TBK1/IRF3-stimulating activity. (**a**) A diagram illustrating domain architectures of wild-type and mutant MAVS. N-terminal CARD: caspase activation and recruitment domain (grey); Region I (blue) and Region II (green) contain putative TRAF-binding motifs; Region III (red) covers amino acids (aa) 401–450; C-terminal TM: transmembrane domain (black). MAVS-(Q145N/E155D), a mutant disrupting two TRAF-binding motifs of Region I; MAVS-(E457D), a mutant disrupting the TRAF-binding motif of Region II; MAVS-(QN2ED) has triple mutations abolishing all three putative TRAF-binding motifs. (**b**–**e**) pcDNA3-UTR-MAVS (wild-type and mutant forms as indicated) together with ISRE-luciferase reporter were transfected into *MAVS−/−* HEK 293T cells. Normal HEK 293T cells were used as a control. Twenty-four hours after transfection, the cells were infected with or without Sendai virus, which were harvested for following analysis 6 h after infection. IFN (**b**), IL-6 (**c**) and ISG54 (**e**) inductions were measured with quantitative PCR (qPCR). (**d**) Cell lysates were separated into S5 and P5 fractions. S5 was examined to detect IRF3 dimerization and the MAVS protein level was examined in P5. The original full blot can be found in Supplementary Fig. 5a. (**f**–**h**) pcDNA3-UTR-MAVS (wild-type and mutant forms as indicated) were transfected into *MAVS−/−* HEK 293T cells. Twenty-four hours after transfection, the cells were infected with or without Sendai virus, which were harvested for following analysis 6 h after infection. ISG54 (**f**), IL-6 (**g**) and IFN (**h**) inductions were measured with qPCR. MAVS protein levels are shown in Supplementary Fig. 1e and Fig. 7a. All data are presented as the mean values based on three independent experiments, and error bars indicate s.d. *P* values were determined by two-tailed Student's *t*-test. *P<0.05 and ***P<0.001. NS indicates no statistically significant difference.

MAVS harbours three putative TRAF-binding motifs in two regions: Region I contains a TRAF2/3/5-binding motif 143-PVQET-147 and a TRAF3/6-binding motif 153-PGENSE-158; Region II includes a TRAF3/6-binding motif 455-PEENEY-460. To investigate how they may contribute to MAVS antiviral function, we generated point mutations disrupting these motifs and examined their respective effects on MAVS antiviral activity (Fig. 1a). Various MAVS forms were expressed at near-physiological levels to avoid spontaneous activation. To prevent the potential involvement of endogenous MAVS, MAVS-deficient (*MAVS−/−*) HEK 293T cells were used for the ectopic expression of MAVS variants (Supplementary Fig. 1a). Wild-type MAVS induced IFN production robustly in response to Sendai virus infection, mimicking a signalling pattern evoked by endogenous MAVS activation (Fig. 1b). In contrast, MAVS mutants (Q145N/E155D and E457D) showed slightly attenuated activity in inducing IFN expression, and triple mutations (QN2ED) abolished MAVS activity completely, suggesting that Regions I and II play redundant roles in antiviral IFN stimulation. Since MAVS activity bifurcates into two branches to coordinately induce IFN expression, that is, activating kinases IKK and TBK1, we next investigated how the above-mentioned mutations affected these two signalling branches specifically. Consistent with a recent report^26^, triple mutations (QN2ED) weakened MAVS activity in inducing interleukin (IL)-6 expression, indicating loss of its activity in stimulating IKK/NF-κB (Fig. 1c). Surprisingly, MAVS-(QN2ED) and the other two mutants remained active in stimulating interferon-stimulated response element (ISRE) reporter, suggesting that they were fully functional in activating the TBK1/IRF3 branch (Supplementary Fig. 1b). Indeed, in response to Sendai virus infection all three mutants induced IRF3 dimerization (Fig. 1d) and expression of ISG54, a direct target of IRF3 transcriptional factor^38^ (Fig. 1e). The attenuated induction of ISG54 by MAVS-(QN2ED) was probably because of lack of IFN induction, which could also induce ISG54 expression. Consistently, MAVS-(QN2ED) induced the phosphorylation of IRF3, a prerequisite for its dimerization and activation (Supplementary Fig. 1c). Therefore, our results suggested that disruption of three previously identified TRAF-binding motifs in Region I and Region II only resulted in the ablation of MAVS activity in stimulating IKK/NF-κB but had no significant effect on its activity in stimulating TBK1/IRF3.

Furthermore, IFN-stimulating defect of MAVS-(QN2ED) could be rescued by co-expression of IKKβ, which is known to activate NF-κB but on its own cannot induce IFN expression (Supplementary Fig. 1d). These data further demonstrated that MAVS contains a TBK1/IRF3-sitmulating region that is independent of its putative TRAF-binding motifs in Regions I and II. We next determined that region aa-401–450 of MAVS, referred to as Region III, was essential for its TBK1/IRF3-stimulating activity. MAVS mutant with deletion of Region III, that is, MAVS-(ΔRegion III), could not induce ISG54 expression with or without Sendai virus infection (Fig. 1f). Instead, it could stimulate NF-κB as much as wild-type MAVS in a signalling-dependent manner (Fig. 1g). The IKK/NF-κB-stimulating activity of MAVS-(ΔRegion III) was abrogated by QN2ED triple mutations, confirming that Region I and Region II were essential for MAVS to stimulate IKK/NF-κB. Consistently, MAVS-(ΔRegion III) could not stimulate IFN expression, suggesting that Region III was indispensable for MAVS antiviral function (Fig. 1h). When Region III of endogenous MAVS was removed (Supplementary Fig. 1f), MAVS could not activate TBK1/IRF3 but was still capable of stimulating IKK/NF-κB in response to Sendai virus infection (Supplementary Fig. 1g).

### Characterization of MAVS Region III

**Figure 2:**
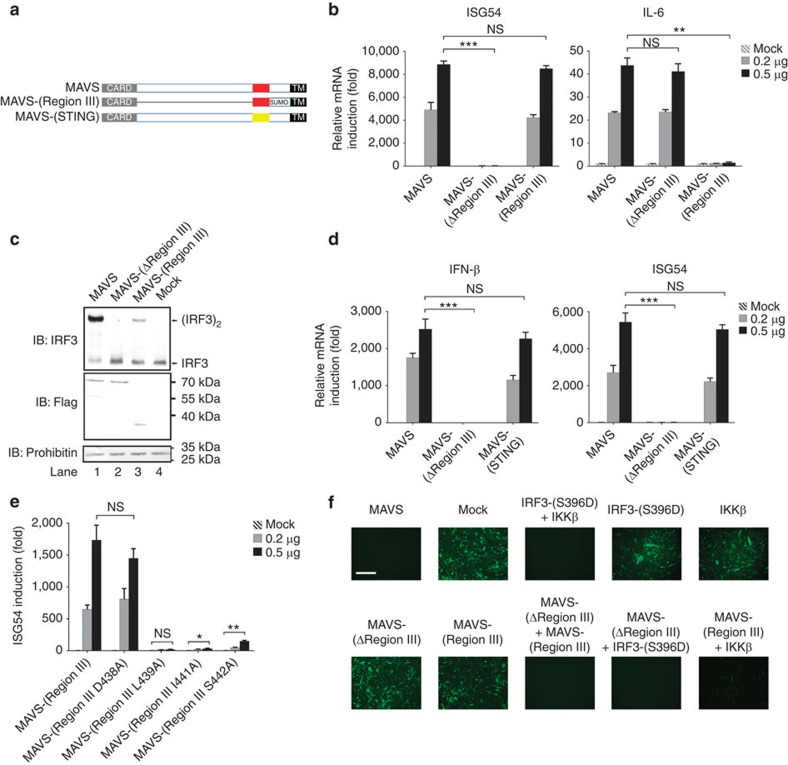
Characterization of the TBK1/IRF3-stimulating region of MAVS. (**a**) A diagram illustrating expression constructs used in Fig. 2. MAVS-(STING) is a chimeric form of MAVS and STING region aa-341–379. MAVS Region III is red and STING region aa-341–379 is yellow. (**b**,**c**) Various amounts of expression constructs on the basis of pcDNA3-flag was transfected into *MAVS−/−* HEK 293T cells. Gene inductions (**b**) were measured 36 h after transfection. IRF3 dimerization (**c**) was also examined. The original full blot can be found in Supplementary Fig. 5b. Protein expression levels are shown in Supplementary Fig. 2c and Fig. 7d. (**d**) Various amounts of pcDNA3-flag-MAVS mutants as indicated were transfected into *MAVS−/−* HEK 293T cells. Gene inductions were measured 36 h after transfection. Protein expression levels are indicated in Supplementary Fig. 2d and Fig. 7e. (**e**) Various amounts of pcDNA3-flag-MAVS-(Region III) mutants were transfected into *MAVS−/−* HEK 293T cells. Gene inductions were measured 36 h after transfection. Protein expression levels are indicated in Supplementary Fig. 2e and Fig. 7f. (**f**) MAVS without Region III is defective in its antiviral function, which could be rescued by co-expression of MAVS (Region III) or a constitutively active form of IRF3-(S396D). Various MAVS forms were transduced into *MAVS−/−* mouse embryonic fibroblast (MEF) cells by retrovirus. Thirty-six hours after transduction, MEF cells were infected with vesicular stomatitis virus (VSV)-ΔM51-GFP. Fluorescent images were taken 24 h after VSV infection to visualize green fluorescent protein (GFP)-positive cells, indicating VSV proliferation. Scale bar, 500 μm. Protein expression levels were detected (Supplementary Fig. 2g and Fig. 8b). All data are presented as the mean values based on three independent experiments, and error bars indicate s.d. *P* values were determined by two-tailed Student's *t*-test. *P<0.05, **P<0.01 and ***P<0.001. NS indicates no statistically significant difference.

To study further MAVS activity in stimulating TBK1/IRF3, we made a construct, namely MAVS-(Region III), containing MAVS N-terminal CARD domain (aa-1–100, to form a prion-like filament)^23^, Region III (aa-401–450), a SUMO moiety (to avoid potential steric hindrance) and MAVS C-terminal TM domain (aa-501–540, to ensure mitochondrial localization)^18^ (Fig. 2a). Expression of MAVS-(Region III) induced ISG54 but not IL-6 expression, suggesting that Region III could activate IRF3 but not NF-κB (Fig. 2b). Consistently, MAVS-(Region III) induced the dimerization of IRF3 (Fig. 2c), confirming that Region III is the TBK1/IRF3-stimulating region of MAVS. On the other hand, IRF3 could be activated by the JAK-STAT signalling pathway, which can be triggered by even trace amount of IFN. To rule out the possibility of potential involvement of IFN and JAK-STAT in analysing the IRF3-stimulating activity of MAVS-(Region III), we generated a double knockout HEK 293T cell line (IFN-associated receptor-deficient *IFNAR2−/−* and MAVS-deficient *MAVS−/−*). After IFN-β treatment, ISRE reporter was activated in *MAVS−/−* 293T cells but not in *IFNAR2−/−* and *MAVS−/−* 293T cells. In contrast, expression of MAVS-(Region III) could activate the ISRE reporter in both *MAVS−/−* and *IFNAR2−/−* and *MAVS−/−* 293T cells, suggesting that MAVS-(Region III) could activate IRF3 independently of the IFN-JAK-STAT pathway (Supplementary Fig. 2a).

Moreover, it was reported that a small region aa-341–379 of STING, an adaptor protein essential for IFN production in response to DNA virus infection, was critical and sufficient for TBK1/IRF3 activation^39^. We replaced Region III of MAVS with the region aa-341–379 of STING to express a chimeric protein, namely MAVS-(STING). Interestingly, MAVS-(STING) was able to induce ISG54 expression (Fig. 2d right), suggesting a similar function of STING region aa-341–379 to MAVS Region III in stimulating TBK1/IRF3. Indeed, MAVS-(STING) induced IFN expression robustly (Fig. 2d left). By analysing amino-acid sequence of MAVS Region III, we identified some residues conserved across species, such as D438, L439, I441, S442 and so on. Mutation of D438 to alanine had no effect on the activity of MAVS-(Region III; Fig. 2e). In contrast, single mutations of L439, I441 and S442 to alanine weakened the TBK1/IRF3-stimulating activity of MAVS to various extents, suggesting that they might be required to form a critical structure for downstream TBK1/IRF3 activation, or post-translational modifications of these residues are required for MAVS activity^40^. Similar results were obtained from these mutants in inducing IRF3 dimerization *in vitro* (Supplementary Fig. 2f).

MAVS-(ΔRegion III) alone could not induce IFN expression, and, as a result, *MAVS−/−* MEF cells ectopically expressing this mutant were more permissive to VSV proliferation than those expressing wild-type MAVS (Fig. 2f), suggesting an essential role of Region III in MAVS antiviral function. Remarkably, the antiviral defect of MAVS-(ΔRegion III) could be rescued by co-expression of MAVS-(Region III) or a constitutively active form IRF3-(S396D), as shown by the inhibition of VSV proliferation, confirming a TBK1/IRF3-stimulating activity of Region III. Consistently, co-expression of MAVS-(Region III) and IKKβ led to robust IFN induction (Supplementary Fig. 2h). On the basis of these results, we concluded that Region III (aa-401–450) is the TBK1/IRF3-stimulating region of MAVS. Similar results were obtained in *MAVS−/−* HEK 293T cells (Supplementary Fig. 2i).

### Identification of inhibitory regions to Region III

**Figure 3:**
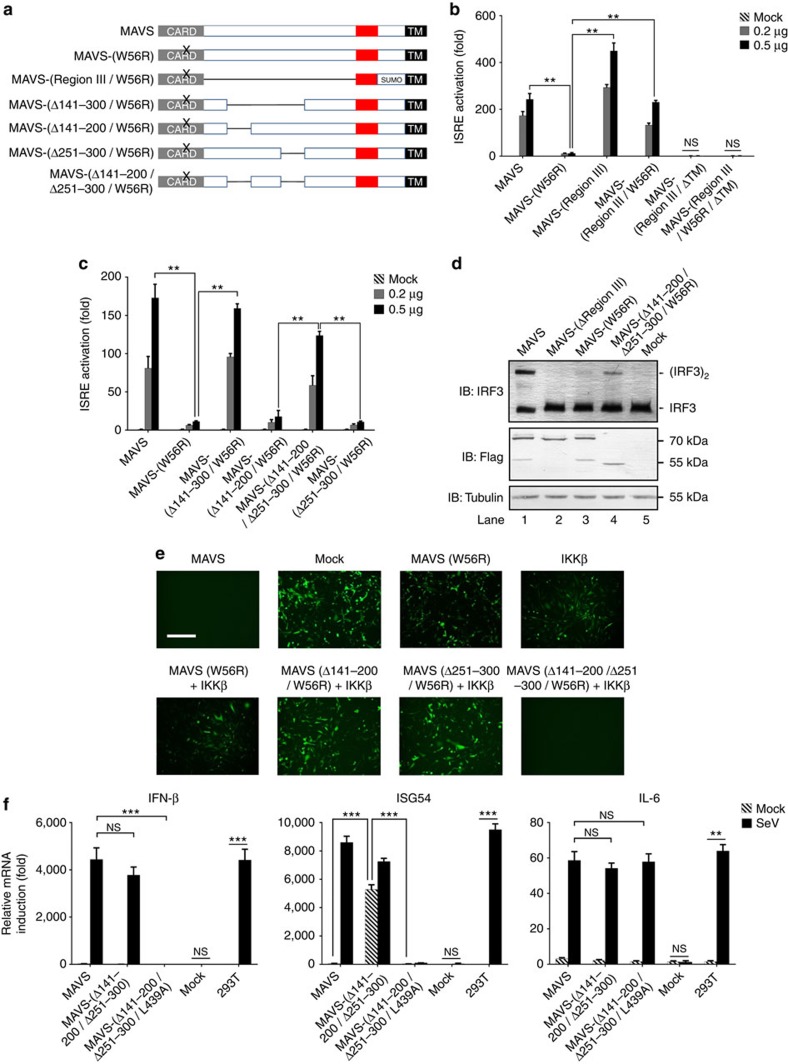
The activity of MAVS Region III is inhibited by leading regions aa-141–200 and aa-251–300 in its quiescent state. (**a**) A diagram illustrating various MAVS deletion mutants to pinpoint inhibitory elements to Region III. W56R: a point mutation replacing W with R. (**b**,**c**) MAVS-(Region III) could stimulate ISRE reporter independently of its filament formation and mapping of inhibitory regions to Region III. Increasing amount of expression vectors as indicated together with ISRE-luciferase reporter construct were transfected into *MAVS−/−* HEK 293T cells and firefly luciferase inductions were measured 36 h after transfection. ΔTM: MAVS with its C-terminal TM domain truncated. Protein expression levels were shown in Supplementary Fig. 3a,b and Fig. 8c,d. (**d**) MAVS-(Δaa-141–200/Δaa-251–300/W56R) could induce IRF3 dimerization. Cell lysates were separated into S5 and P5 fractions. S5 was examined to detect IRF3 dimerization and protein expression level was examined. The original full blot can be found in Supplementary Fig. 5c. (**e**) *MAVS−/−* MEF cells were transduced with various MAVS forms as indicated followed by VSV-ΔM51-GFP infection. Fluorescent images were taken 24 h after virus infection to examine VSV proliferation. Scale bar, 500 μm. Protein expression levels were indicated in Supplementary Fig. 3g and Fig. 9a. (**f**) pcDNA3-UTR-MAVS (wild-type and mutant forms as indicated) was transfected into *MAVS−/−* HEK 293T cells. Twenty-four hours after transfection, the cells were infected with or without Sendai virus, which were harvested for following analysis 6 h after infection. IFN (left), ISG54 (middle) and IL-6 (right) inductions were measured with qPCR. Protein expression levels were shown in Supplementary Fig. 3h and Fig. 9b. All data are presented as the mean values based on three independent experiments, and error bars indicate s.d. *P* values were determined by two-tailed Student's *t*-test. **P<0.01 and ***P<0.001. NS indicates no statistically significant difference.

Having identified Region III as the TBK1/IRF3-stimulating region, we next investigated the mechanism regulating its activity. MAVS activity is regulated by its prion-like filament formation and recent structural study showed that an amino-acid residue W56 is critical for the MAVS CARD domain to form the prion-like filament^25^. Indeed, a point mutation (W56R) abolished full-length MAVS activity in activating IRF3 (Fig. 3b). In contrast, the same point mutation in MAVS-(Region III) did not abrogate its activity, suggesting that the TBK1/IRF3-stimulating activity of Region III was not dependent on MAVS filament *per se* and we further hypothesized that there was an inhibitory element in full-length MAVS-(W56R) to keep itself inactive. We also noticed that the activity of MAVS-(Region III) was dependent on its TM domain, confirming the previous report that MAVS antiviral function requires its mitochondrial localization^18^.

To identify the inhibitory element to Region III, we made a series of deletion mutants of MAVS and examined their activity in activating ISRE-luciferase reporter. We included the point mutation W56R in all MAVS deletion mutants to prevent activation due to spontaneous oligomerization of the CARD domain. Strikingly, when region aa-141–300 was deleted, MAVS showed potent IRF3-stimulating activity, suggesting that region aa-141–300 plays an inhibitory role to Region III (Fig. 3c). Further studies showed that deletion of two separate segments in region aa-141–300 (Δaa-141–200 and Δaa-251–300) led to robust MAVS activity in stimulating ISRE reporter. Deletion of either aa-141–200 or aa-251–300 alone did not lead to MAVS activation in stimulating ISRE, suggesting a two-layered inhibition to Region III. Consistently, MAVS (Δaa-141–200/Δaa-251–300/W56R) could induce the dimerization of IRF3 in cells (Fig. 3d) and *in vitro* (Supplementary Fig. 3c). In addition, the CARD domain was dispensible for MAVS-(Δaa-141–300/W56R) to activate TBK1-IRF3 (Supplementary Fig. 3d). Furthermore, co-expression of MAVS (Δaa-141–300/W56R) and IKKβ induced the expression of IFN (Supplementary Fig. 3f) and rendered *MAVS−/−* MEF cells resistant to VSV replication (Fig. 3e).

Endogenous MAVS does not show TBK1/IRF3-stimulating activity until receiving upstream signal to form prion-like filaments. The inhibitory regions we identified likely underlie the autoinhibitory mechanism preventing the activity of Region III in unstimulated MAVS. To study the inhibitory elements identified in a context of virus infection, MAVS was expressed at a near-physiological level. Full-length MAVS could only induce ISG54 expression in response to Sendai virus infection, while MAVS (Δaa-141–300) induced ISG54 expression regardless of virus infection (Fig. 3f). The activity of MAVS (Δaa-141–300) was abolished by a single point mutation L439A, confirming the essential role of Region III in stimulating TBK1/IRF3.

One possible mechanism for region aa-141–300 to inhibit Region III is that region aa-141–300 may serve as a platform to recruit another protein to inhibit Region III. Our results showed that two segments aa-141–200 and aa-251–300 could inhibit Region III independently (Fig. 3c), and we reasoned that it is unlikely for two separate fragments to recruit the same ‘inhibitory protein'. More importantly, recombinant MAVS protein without inhibitory region (GST-MAVS-(aa-401–540)), which was expressed in *Escherichia coli* (*E. coli)*, was active in inducing IRF3 dimerization. In contrast, recombinant MAVS with inhibitory region (GST-MAVS-(aa-151–540)) was not active (Supplementary Fig. 3i). These results further supported that region aa-151–300 directly inhibits Region III, rather than through recruiting other inhibitory proteins from the host cells. We next investigated whether MAVS region aa-151–300 could inhibit Region III *in trans*. When region aa-151–300 and MAVS-(Region III/W56R) were co-expressed as individual proteins in cells, we could not detect the inhibitory effect of region aa-151–300 to Region III, suggesting that region aa-151–300 could not inhibit the activity of Region III *in trans* (Supplementary Fig. 3j).

### Characterization of MAVS Region I and II

**Figure 4:**
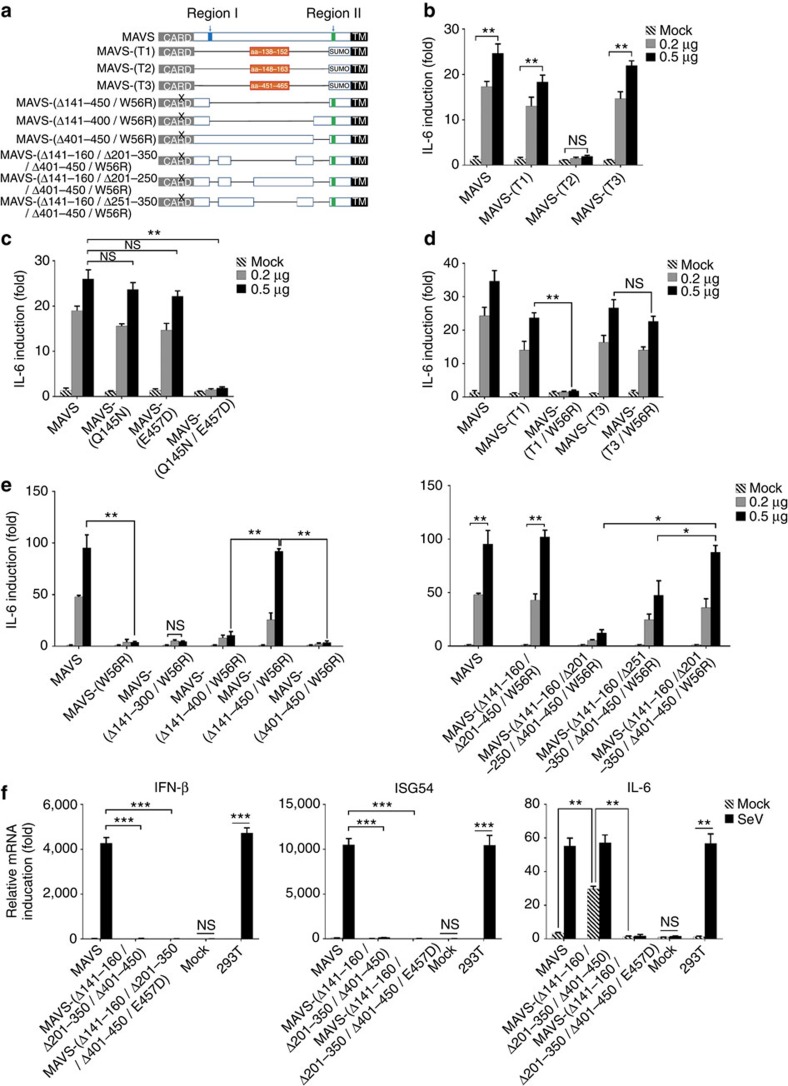
Two NF-κB-stimulating regions of MAVS are regulated by different mechanisms. (**a**) A diagram illustrating various MAVS-mutant forms used in Fig. 4. MAVS-(T1): encompassing amino acids 138–152; MAVS-(T2): encompassing amino acids 148–163; MAVS-(T3): encompassing amino acids 451–465. (**b**–**e**) Increasing amount of expression vectors as indicated transfected into *MAVS−/−* HEK 293T cells and IL-6 inductions were measured 36 h after transfection. (**b**) Two alternative regions of MAVS (aa-138–152 and aa-451–465) were responsible for NF-κB activation. Protein expression levels were indicated in Supplementary Fig. 4a and Fig 9e. (**c**) MAVS with double mutations (Q145N/E457D) lost its activity in stimulating IL-6 expression. Protein expression levels were shown in Supplementary Fig. 4b and Fig 9f. (**d**) MAVS Region II could activate NF-κB independently of its filament formation. Protein expression levels were indicated in Supplementary Fig. 4e and Fig. 10a. (**e**) Regions aa-201–350 and aa-401–450 inhibit the activity of Region II in quiescent MAVS. Protein expression levels were shown in Supplementary Fig. 4h and Fig. 10c,d. (**f**) pcDNA3-UTR-MAVS (wild-type and mutant forms as indicated) was transfected into *MAVS−/−* HEK 293T cells. Twenty-four hours after transfection, the cells were infected with or without Sendai virus, which were harvested for following analysis 6 h after infection. IFN (left), ISG54 (middle) and IL-6 (right) inductions were measured with qPCR. Protein expression levels were indicated in Supplementary Fig. 4k and Fig. 10f. All data are presented as the mean values based on three independent experiments, and error bars indicate s.d. *P* values were determined by two-tailed Student's *t*-test. *P<0.05, **P<0.01 and ***P<0.001. N.S. indicates no statistically significant difference.

As shown in Fig. 1c, MAVS-(QN2ED) lost its NF-κB-stimulating activity, suggesting the essential role of previously identified TRAF-binding motifs. To further define the NF-κB-stimulating activity of MAVS, we made expression vectors containing these putative TRAF-binding motifs flanked by MAVS CARD and TM domains, referred to as MAVS-(T1), MAVS-(T2) and MAVS-(T3), respectively (Fig. 4a). Both MAVS-(T1) and MAVS-(T3) potently induce IL-6 expression, suggesting that aa-138–152 and aa-451–465 are IKK/NF-κB-stimulating regions for MAVS (Fig. 4b). In contrast, MAVS-(T2) showed no NF-κB-stimulating activity. To validate the result, we examined the effect of mutations disrupting motifs 143-PVQET-147 and 455-PEENEY-460 on MAVS activity. Strikingly, while single mutation Q145N or E457D barely affected NF-κB-stimulating activity of full-length MAVS, double mutations (Q145N/E457D) abolished its activity (Fig. 4c), showing a similar effect to triple mutations (QN2ED). These results suggested that Region I and Region II play redundant roles in stimulating NF-κB, whereas previously identified TRAF-binding motif 153-PGENSE-158 does not harbour any NF-κB-stimulating activity. Consistently, expression of either MAVS-(T1) or MAVS-(T3) could induce IFN expression when complemented with the constitutively active form of IRF3-(S396D) (Supplementary Fig. 4c) and rendered *MAVS−/−* MEF cells resistant to VSV replication (Supplementary Fig. 4d). We concluded that Region I (aa-138–152) and Region II (aa-451–465) provided MAVS with NF-κB-stimulating activity redundantly.

### Regulation of the activity of Regions I and II

We next investigated the mechanism by which the NF-κB-stimulating activity of MAVS is regulated. We first examined whether the activity of MAVS-(T1) and MAVS-(T3) in stimulating NF-κB is dependent on MAVS filament formation. Although MAVS-(T1) showed robust activity, MAVS-(T1/W56R) lost its activity in stimulating NF-κB, suggesting that MAVS filament formation is essential for the Region I to activate NF-κB, which may facilitate Region I to form a specific conformation (Fig. 4d). Moreover, neither deletion of the CARD domain or region aa-101–140 from MAVS-(W56R) led to IL-6 induction, indicating that the lack of NF-κB-stimulating activity from MAVS-(T1/W56R) is not because of the inhibitory effect by the CARD domain or region aa-101–140 (Supplementary Fig. 4f). In contrast, MAVS-(T3/W56R) was as active as MAVS-(T3) in stimulating NF-κB, suggesting that its activity is independent of the filament formation. We hypothesized that there was an inhibitory mechanism masking the activity of Region II in unstimulated MAVS, in a manner similar to the inhibition of Region III by region aa-141–300 as described above.

To identify the potential inhibitory element to Region II, we made a series of deletion on full-length MAVS and examined their activity in stimulating NF-κB. Once again we included the point mutation W56R in all MAVS deletion mutants to prevent spontaneous filament formation. First, when region aa-141–450 was deleted, MAVS showed robust NF-κB-stimulating activity, suggesting that region aa-141–450 plays an inhibitory role on Region II in unstimulated MAVS (Fig. 4e left). In contrast, MAVS-(Δaa-141–400/W56R) did not show any NF-κB-stimulating activity, indicating that Region III played an inhibitory role on Region II. However, deletion of Region III (MAVS-(Δaa-401–450/W56R)) alone did not lead to its NF-κB-stimulating activity, suggesting that there may be another layer of inhibitory effect from region aa-141–400. We then found that region aa-201–350 conferred the second layer of inhibition on Region II, as MAVS-(Δaa-141–160/Δaa-201–350/Δaa-401–450/W56R) stimulated NF-κB robustly (aa-141–160 was omitted to avoid the potential contribution of Region I; Fig. 4e right). In addition, the CARD domain was not required for MAVS-(Δaa-141–160/Δaa-201–350/Δaa-401–450) to activate NF-κB (Supplementary Fig. 4i).

To investigate the above-mentioned inhibitory elements in a context of RLR activation, we generated a deletion mutant of MAVS (Δaa-141–160/Δaa-201–350/Δaa-401–450) with intact CARD domain and expressed it at a near-physiological level (Fig. 4f). Wild-type MAVS could only induce IL-6 expression in response to Sendai virus infection, while MAVS (Δaa-141–160/Δaa-201–350/Δaa-401–450) induced IL-6 expression independently of virus infection. Furthermore, the NF-κB-stimulating activity of MAVS (Δaa-141–160/Δaa-201–350/Δaa-401–450) was abrogated by a point mutation E457D, confirming the inhibitory role of regions aa-201–350 and aa-401–450 to Region II in resting MAVS.

### MAVS Regions I, II and III recruit preferential TRAFs

**Figure 5:**
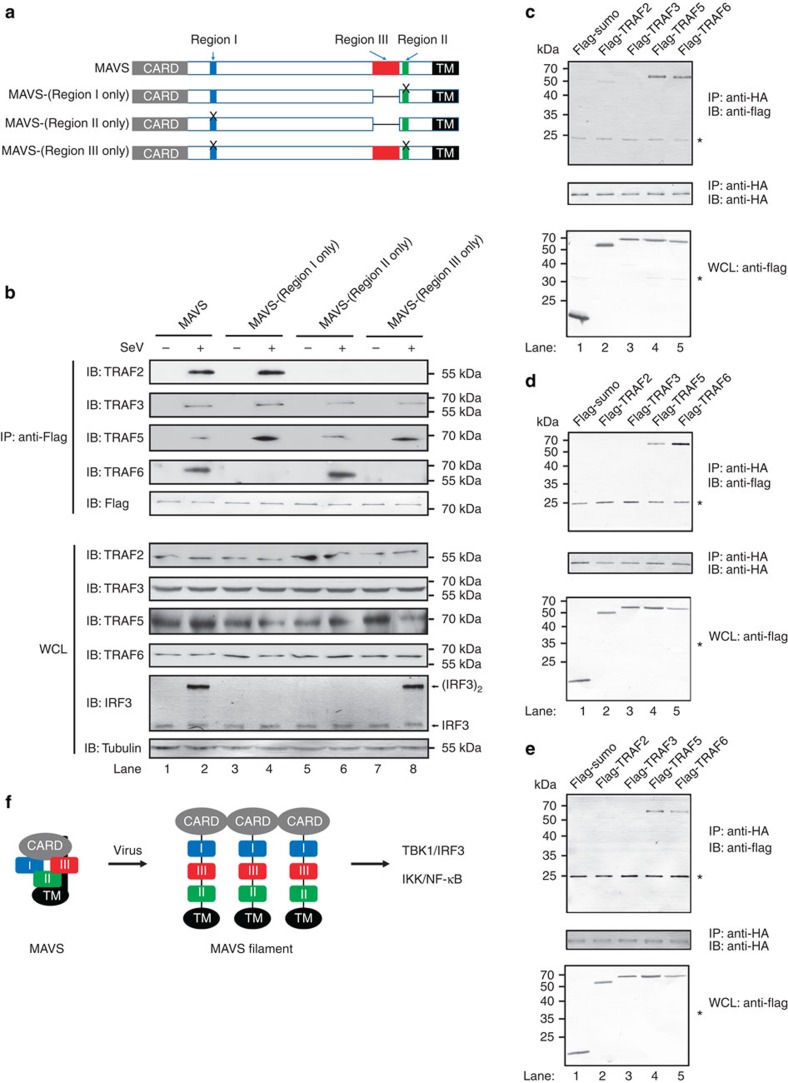
Regions I, II and III of MAVS recruit TRAFs with differential preference. (**a**) A diagram illustrating various MAVS constructs. MAVS-(Region I only), a point mutation E457D and a deletion of Region III; MAVS-(Region II only), two point mutations Q145N/E155D and a deletion of Region III; MAVS-(Region III only), three point mutations Q145N/E155D/E457D. (**b**) pcDNA3-UTR-flag-MAVS (wild-type and mutant forms as indicated) were transfected into *MAVS−/−* HEK 293T cells. Twenty-four hours after transfection, the cells were infected with or without Sendai virus, which were harvested for following co-immunoprecipitation with M2 beads 12 h after infection. Immunoblotting was performed with antibodies as indicated for IP products and whole-cell lysate (WCL). The original full blot can be found in Supplementary Fig. 5d. (**c**–**e**) Regions I, II and III of MAVS bind to TRAFs with different preference. Constructs MAVS-(T1) (**c**), MAVS-(T3) (**d**) and MAVS-(Region III) (**e**) were transfected into *MAVS−/−* HEK 293T cells to express HA-tagged Regions I, II and III, respectively, which were isolated with anti-HA agarose beads and then used to pull down various flag-tagged TRAFs. Immunoblotting was then performed. Asterisk indicated nonspecific bands. The original full blot can be found in Supplementary Fig. 6a–c. (**f**) A working model showing autoinhibitory mechanisms modulating MAVS antiviral activity. MAVS is composed of three active regions: NF-κB-stimulating Region I and Region II and the IRF3-stimulating Region III. In unstimulated cells, Region I (blue) is in an inactive conformation; Region II (green) and Region III (red) are inhibited by their respective adjacent regions (black curve lines). On viral infection, RIG-I/double-stranded RNA/ub complex induces MAVS to form prion-like filaments through its CARD domain (grey). Consequently, Region I becomes active probably by adopting a particular conformation, while Region II and Region III are released from autoinhibition to activate downstream signalling molecules including TBK1/IRF3 and IKK/NF-κB.

Numerous reports have shown that multiple TRAFs are involved in MAVS-mediated antiviral signalling, presumably playing redundant roles. In particular, TRAF2 and TRAF6 were reported to comigrate with the MAVS filament (the active form) on sucrose gradient ultracentrifugation; however, how they contributed to MAVS activity in stimulating TBK1/IRF3 and IKK/NF-κB, respectively, remained unclear^23^. As we found that Regions I, II and III harbour distinct activities, we next examined how TRAFs might contribute to MAVS activity by association with Regions I, II and III. In response to Sendai virus infection, TRAF2/3/5/6 were all pulled down by wild-type MAVS (Fig. 5b). Interestingly, MAVS-(Region I only), but not MAVS-(Region II only) or MAVS-(Region III only), bound to TRAF2 after Sendai virus infection. In contrast, MAVS-(Region II only), but not MAVS-(Region I only) or MAVS-(Region III only), bound to TRAF6. Moreover, these active regions bound to TRAF3/5 with no preference. Collectively, these data indicated that Regions I, II and III recruited TRAF2/3/5/6 differentially and in a signalling-dependent manner.

In light of that TRAFs might be expressed at variable levels in various tissue or cell types, we next investigated whether TRAFs, when expressed at the same level, could be recruited to three active regions of MAVS differentially. These three regions were then tagged with haemagglutinating (HA) epitope and flanked with N-terminal CARD and C-terminal TM domains, which were then used as a bait to pull down flag-tagged TRAF2/3/5/6. As shown in Fig. 5c,d,e, TRAF5 and TRAF6 were pulled down by the three regions to different extents while the control protein Flag-sumo was not detected in immunoprecipitation (IP) products. Interestingly, TRAF2 was pulled down only by Region I but not by Region II, suggesting that TRAF2 might be involved in IKK/NF-κB activation. Meanwhile, there was a weak interaction between TRAF2 and Region III, suggesting that it might be also involved in TBK1/IRF3 activation. Under this condition, we could not observe interaction between TRAF3 and Regions I, II or III, probably because of the nature of ectopically expressed TRAF3 and we cannot rule out the possibility that TRAF3 might bind to other regions of MAVS to facilitate interferon production.

## Discussion

In this report, we identified a novel region of MAVS responsible for TBK1/IRF3 activation and defined two regions for IKK/NF-κB activation. We further revealed a multilayered autoinhibitory mechanism modulating MAVS activity in the absence of stimulation. The autoinhibition can be released by MAVS prion-like filament formation. On the basis of these results, we propose a working model for the regulation of MAVS antiviral activity (Fig. 5f).

MAVS has a CARD and a TM domain, which are essential for its antiviral function. In addition, the region in between contains three putative TRAF-binding motifs, presumably responsible for MAVS to activate downstream signalling molecules. Disruption of these motifs by point mutation was shown to abrogate its activity in stimulating TBK1/IRF3 and IKK/NF-κB (ref. 26). In contrast, we found that these mutations only impaired MAVS activity in stimulating IKK/NF-κB, whereas activation of TBK1/IRF3 was not affected. We went further to demonstrate that Region I (aa-138–152) and Region II (aa-451–465) harbour IKK/NF-κB-stimulating activity independently, while the putative TRAF-binding motif 153-PGENSE-158 may be dispensable for MAVS activity. More importantly, we identified MAVS Region III (aa-401–450) as a novel essential region that is responsible for TBK1/IRF3 stimulation. While we were preparing the manuscript, a report showed that the 438-DLAIS-442 motif in MAVS is critical for its function^41^. Although this motif is a part of MAVS Region III, it is not sufficient in stimulating TBK1/IRF3 and our data showed that the conserved aspartic acid residue at position 438 (human MAVS) was dispensable for the activity of Region III. Instead, we found that L439, I441 and S442 were critical residues for MAVS activity. Collectively, we provided both loss-of-function and gain-of-function evidence to demonstrate that the entire MAVS Region III is required for TBK1/IRF3 activation.

Despite many studies, the molecular mechanism underlying the activation of IKK and TBK1 by MAVS remains mysterious, in which multiple TRAFs might be involved. To start to unravel these questions, it is critical to identify active regions in MAVS and assign them the specific functions. Our data indicated that Regions I and II might recruit TRAF2 and TRAF6, respectively, to activate IKK/NF-κB. Meanwhile, TRAF3/5 could be recruited by all three regions to activate IKK/NF-κB or TBK1/IRF3. This result suggested that these active regions of MAVS may determine the substrate specificity for TRAFs. The specific activation of TRAFs could avoid accidental activation of other irrelevant pathways, such as TNF- and IL-1-mediated signalling pathways, in which both TRAF5 and TRAF6 are also involved. Furthermore, we identified two inhibitory regions to Region III in unstimulated MAVS, but were unable to detect their interactions by co-immunoprecipitation of the two regions expressed as separate proteins. It is possible that the sought-for interactions can only occur in *cis* rather than in *trans*, which is in consistence with our data showing that region aa-151–300 can only inhibit Region III in *cis* but not in *trans*. The same scenario applied to Region II and its inhibitory regions. Future structural study may reveal the inhibitory mechanism at the molecular level, which should shed light into antiviral therapeutic development.

An autoinhibitory mechanism in RIG-I has been shown to prevent its spontaneous activation. The C-terminal regulatory domain and middle helicase domain of RIG-I sequester its 2CARD domains before virus infection. The inhibition is released by binding of base-paired RNA to RIG-I, which then activates downstream adaptor protein MAVS. As a result, MAVS forms prion-like filaments in a self-propagating manner, with the potential to amplify signal rapidly. The autoinhibitory mechanisms uncovered in this study serve as an important ‘safety check' to prevent spontaneous signalling from MAVS. Given the importance of the RIG-I/MAVS pathway, it is critical to keep them in the quiescent state in the absence of virus, but trigger their activation rapidly on viral infection. These requirements are met by the elaborate autoinhibition and activation mechanisms of RIG-I and MAVS.

## Methods

### Plasmids and antibodies

Human complementary DNA was prepared from total RNA extracted from HEK 293T cells. MAVS cDNA containing 5′-untranslated region (UTR) was amplified using two primers (5′-CCCAAGCTTACATGGCCAATGGCCGCG-3′ and 5′-AAATCTAGACTAGTGCAGACGCCGCCGGTACAGCACC-3′) and was then cloned into pcDNA3.0 vector between the restriction enzyme sites of HindIII and XbaI (namely pcDNA3-UTR-MAVS). pcDNA3-flag-MAVS was as described^23^. Human cDNA open reading frames encoding TRAF2/3/5/6, IRF3 S396D and IKKβ were cloned into pcDNA3-flag expression vector and primers used for amplification can be found in Supplementary Table 1. For retrovirus-mediated transduction, N-terminally Flag-tagged MAVS (wild-type and mutants), IRF3 (S396D) and IKKβ were cloned into the expression vector pMX between restriction enzyme sites of BamHI and NotI and primers used for amplification can be found in Supplementary Table 1. To express MAVS recombinant protein in *E. coli*, MAVS fragments were cloned into the pET-28a vector^23^ between restriction enzyme sites of BamHI and XhoI with N-terminal His_6_-SUMO or His_6_-GST tag. Constructs with mutation or deletion were made with the Fast-mutagenesis Kit (TransGen Biotech, Beijing, China), QuikChange Lightning Multi Site-Directed Mutagenesis Kit (Stratagene) or overlapping PCR strategy and primers used for amplification can be found in Supplementary Table 1. All constructs were confirmed using DNA sequencing. Antibody against MAVS was raised by immunizing rabbits with the recombinant protein SUMO-MAVS-(aa-301–510) and used at a dilution of 1:10,000; Antibodies against Flag tag (F3165, F7425, dilution 1:5,000) and tubulin (T5168, dilution 1:7,500) were purchased from Sigma; HA antibody (AP1012a, dilution 1:5,000) was from Abgent; antibody against IRF3 (2241-1, dilution 1:3,000) was from Epitomics; pIRF3 S396 antibody (MA5–14917, dilution 1:1,000) was from Pierce; antibody against phosphor-STAT1 (#7649, dilution 1:1,000) was from Cell Signaling Technology; Antibodies against TRAF2 (SC-8760, dilution 1:1,000), TRAF3 (SC-1828, dilution 1:1,000) and TRAF6 (SC7221, dilution 1:1,000) were from Santa Cruz; antibodies against TRAF5 (ab12123, dilution 1:2,000) and prohibitin (ab75766, dilution 1:10,000) were from Abcam.

### Cells and viruses

*MAVS−/−* MEF cells as described^23^ were cultured in DMEM medium supplemented with FBS (fetal bovine serum; 10%, ExCell Bio, FSP500), penicillin (100 U ml^−1^) and streptomycin (100 μg ml^−1^). HEK 293T cells were as described^23^ and were grown in DMEM medium supplemented with 10% calf serum and antibiotics. Recombinant virus VSV-ΔM51-GFP (ref. 42) was amplified in Vero cells^18^ and used with a multiplicity of infection=1. For VSV infection, *MAVS−/−* MEF cells were seeded in 12-well plates at a density of 1 × 10^5^ cells per well. Before incubation with 0.5 ml VSV in DMEM medium MEF cells were washed once with PBS. One hour after incubation, equal volume of DMEM with 20% FBS and antibiotics was added to the cells. Twenty-four hours after infection, cells were harvested for following analysis. Sendai virus (Cantell strain) was from Charles River Laboratories and used at a concentration of 100 HA units ml^−1^.

### Generation of *MAVS−/−* HEK 293T cells

MAVS-deficient HEK 293T cells were generated by transcription activator-like effector nucleases (TALEN)-mediated genome engineering. Two TALEN constructs targeting sequences (5′-ACAACAGCTGCAGAGAGA-3′ and 5′-GGGTCTCCTGGACAGGCA-3′, respectively) in the third exon of the human *MAVS* gene were made using the FastTALE™TALEN Assembly Kit (Sidansai Biotechnology, Shanghai, China). TALEN-targeting constructs were transfected into HEK 293T cells using Lipofectamine 2000 (Invitrogen). Individual cellular colonies were picked up 2 weeks after transfection and genomic DNAs were then extracted and sequenced to verify modification on the *MAVS* gene. A reading frameshift in the *MAVS* gene after amino acid P138 was introduced in the *MAVS−/−* HEK 293T cells, thus generating a non-functional MAVS product. For generation of HEK 293T cell line with knockout of MAVS Region III (*MAVS(aa-401–450)−/−*), two TALEN constructs targeting sequences (5′-CAGTGCCAGCACCTCCT-3′ and 5′-CATTCTCCTCTGGGCCAT-3′) in the seventh exon of the *MAVS* gene were made. To generate HEK 293T cell line deficient in the interferon-alpha/beta receptor beta chain (*IFNAR2*−/−) in the context of MAVS knockout, we adopted the CRISPR/Cas9 technique and designed a sgRNA targeting the sequence (5′-CATCTTCAGATCACTTAATT-3′) in the first exon of human *IFNAR2*. The CRISPR/Cas9 vector pX330-puro was kindly provided by Dr Yan Zhang (Institut Pasteur of Shanghai, Chinese Academy of Sciences). This vector was modified by insertion of a puromycin N-acetyl-transferase gene into a commercial plasmid pX330-U6-Chimeric_BB-CBh-hSpCas9 ( http://www.addgene.org/42230/).

### Luciferase reporter assay

HEK 293T cells were seeded in 12-well plates at a density of 1 × 10^5^ cells per well and co-transfected with 20 ng of reporter gene (IFN-luciferase, ISRE-luciferase or NF-κB-luciferase)^18^, 20 ng of pCMV-LacZ (ref. 18) as internal control, and indicated expression vectors by calcium phosphate method. Thirty-six hours after transfection, cells were harvested and lysed in Passive Lysis Buffer (Promega). Firefly luciferase activities were measured with luminometer using the Luciferase Reporter Kit (Promega) and LacZ activities were measured by o-Nitrophenyl-β-D-Galactopyranoside (ONPG) assay in buffer (200 mM Na_2_HPO_4_/NaH_2_PO_4_ pH 7.3, 2 mM MgCl_2_, 100 mM β-mercaptoethanol, 1.33 mg ml^−1^ ONPG) following a protocol provided by Sigma Technical Bulletin (GALA-1KT). Fold induction of firefly luciferase was normalized to LacZ activity. Data were expressed as fold induction over empty vector-transfected controls.

### Quantitative PCR

Total RNA was isolated using the RNAsimple total RNA kit (Tiangen, Shanghai, China) from HEK 293T or MEF cells, followed by reverse transcription into cDNAs using the iScript Kit (Bio-rad). cDNAs were then used as template for qPCR assay using iTaq universal SYBR green supermix (Bio-rad). For HEK 293T cells, GAPDH was used as internal control and β-actin was used as internal control for *MAVS−/−* MEF cells. Induction fold of each experiments was determined with the ΔΔ*C*q method and quantitative PCR (qPCR). Accuracy was determined using the melt curve test. Primers used to amplify specific genes are listed as following:

Human GAPDH 5′-AGAAGGCTGGGGCTCATTTG-3′ and 5′-AGGGGCCATCCACAGTCTTC-3′; Human IFN-β 5′-CAGCAGTTCCAGAAGGAGGA-3′ and 5′-AGCCAGGAGGTTCTCAACAA-3′; Human IL-6 5′-GAGAAAGGAGACATGTAACAAGAGTAAC-3′ and 5′-ACTCATCTGCACAGCTCTGGC-3′; Human ISG54 5′-CTGAACCGAGCCCTGCCGAAC-3′ and 5′-GCTGCCTCGTTTTGCCCTTTGAG-3′.

### Retrovirus packaging and transduction

The retrovirus-based expression vector, pMX-Flag, and packaging cell line^43^ were provided by Dr Lijian Hui (Shanghai Institute of Biochemistry and Cell Biology). Expression vectors were transfected into HEK 293T packaging cell line using Lipofectamine 2000 (Invitrogen) and the retrovirus-containing culture medium was collected 24 h after transfection. For retrovirus-mediated transduction, *MAVS−/−* MEF cells were seeded in 12-well plates at a density of 1 × 10^5^ cells per well and washed once with PBS buffer before incubation with 0.25 ml retrovirus in the presence of 8 ng μl^−1^ polybrene, which were replaced by fresh culture medium containing 5% FBS 12 h after transduction. Cells were harvested for qPCR assay or western blot analysis 36 h after transduction.

### Statistical analysis

All data are presented as mean values±s.d. on the basis of three independent experiments. Statistical significance between two groups was determined by two-tailed Student's *t*-test, and a *P* value of less than 0.05 was considered statistically significant.

### Immunoprecipitations

*MAVS−/−* HEK 293T cells were transfected with indicated constructs. Twenty-four hours after transfection, cells were washed once with PBS buffer before being resuspended with lysis buffer (HEPES (pH 7.5) 20 mM, MgCl_2_ 5 mM, KCl 10 mM, EGTA 0.5 mM, Triton X-100 1% and protease inhibitor cocktail (Roche)). Following a brief centrifugation, anti-flag M2 affinity gel (Sigma A2220) or anti-HA agarose beads (Thermo 26181) were incubated with cell lysate for 4 h at 4 °C. M2 beads were then spun down and washed three times with lysis buffer before immunoblotting analysis. HA beads were spun down and incubated with cell lysate containing various Flag-tagged TRAFs (20 ng μl^−1^). Two hours after incubation, HA beads were spun down and washed for three times with lysis buffer followed by SDS–PAGE separation and western immunoblot analysis.

### IRF3 dimerization analysis

HEK 293T cells were fractionated into S5 and P5 as described^23^. Briefly, HEK 293T cells were homogenized in hypotonic buffer (10 mM Tris-Cl (pH 7.5), 10 mM KCl, 0.5 mM EGTA and 1.5 mM MgCl_2_), and the homogenates were then separated into S5 and P5 fractions by centrifugation. S5 was then separated on native PAGE gel followed by western blot analysis. P5 was used for IRF3 dimerization assay *in vitro* as described^23^ except that *in vitro*-translated IRF3 was omitted and endogenous IRF3 was detected by western blot analysis.

### Fluorescence microscopy

Various MAVS forms as specified were transduced into *MAVS−/−* MEF cells with retrovirus. Thirty-six hours after transduction, cells were infected with VSV-ΔM51-GFP. Twenty-four hours later, fluorescent images were taken from live cells using Olympus IX71 inverted fluorescence microscope. For imaging with HEK 293T cells, images were taken 8 h after VSV infection.

### Recombinant protein preparation

The bacterial expression vector pET-28a-His-Sumo-MAVS-(aa-301–510), pET-28a-His-GST-MAVS-(aa151–450) or pET-28a-His-GST-MAVS-(aa-401–450) were transformed in BL-21 strain (TransGen Biotech). Recombinant proteins were induced with 0.2 mM isopropyl-b-D-thiogalactoside at 20 °C for 12 h. After sonication in lysis buffer (10 mM Tris-Cl (pH 8.0), 0.5 M NaCl, 5 mM β-mercaptoethanol (BME), 0.5 mM dithiothreitol, 5% Glycerol and 0.5 mM phenylmethylsulphonyl fluoride), cell lysates were centrifuged at 50,000*g* for 30 min. Recombinant proteins Sumo-MAVS-(aa-301–510), GST-MAVS-(aa-151–540) and GST-MAVS-(aa-401–540) were purified with Ni-NTA agarose beads according to the manufacturer's protocol (Qiagen, 30210). Sumo-MAVS-(aa-301–510) was used as the antigen to make MAVS antibody. GST-MAVS-(aa-151–540) and GST-MAVS-(aa-401–540) were used in IRF3 dimerization analysis.

## Additional information

**How to cite this article:** Shi, Y. *et al*. An autoinhibitory mechanism modulates MAVS activity in antiviral innate immune response. *Nat. Commun*. 6:7811 doi: 10.1038/ncomms8811 (2015).

## Supplementary Material

Supplementary Information Supplementary Figures 1-10 and Supplementary Table 1
