# Supplementary material for: An autoinhibitory mechanism modulates MAVS activity in antiviral innate immune response
Source: Nat Commun. 2015 Jul 17;6:7811. doi: 10.1038/ncomms8811 (PMC4518314; doi:10.1038/ncomms8811)
Supplement: Supplementary Information — Supplementary Figures 1-10 and Supplementary Table 1 [file ncomms8811-s1.pdf]

Supplementary Figure 1

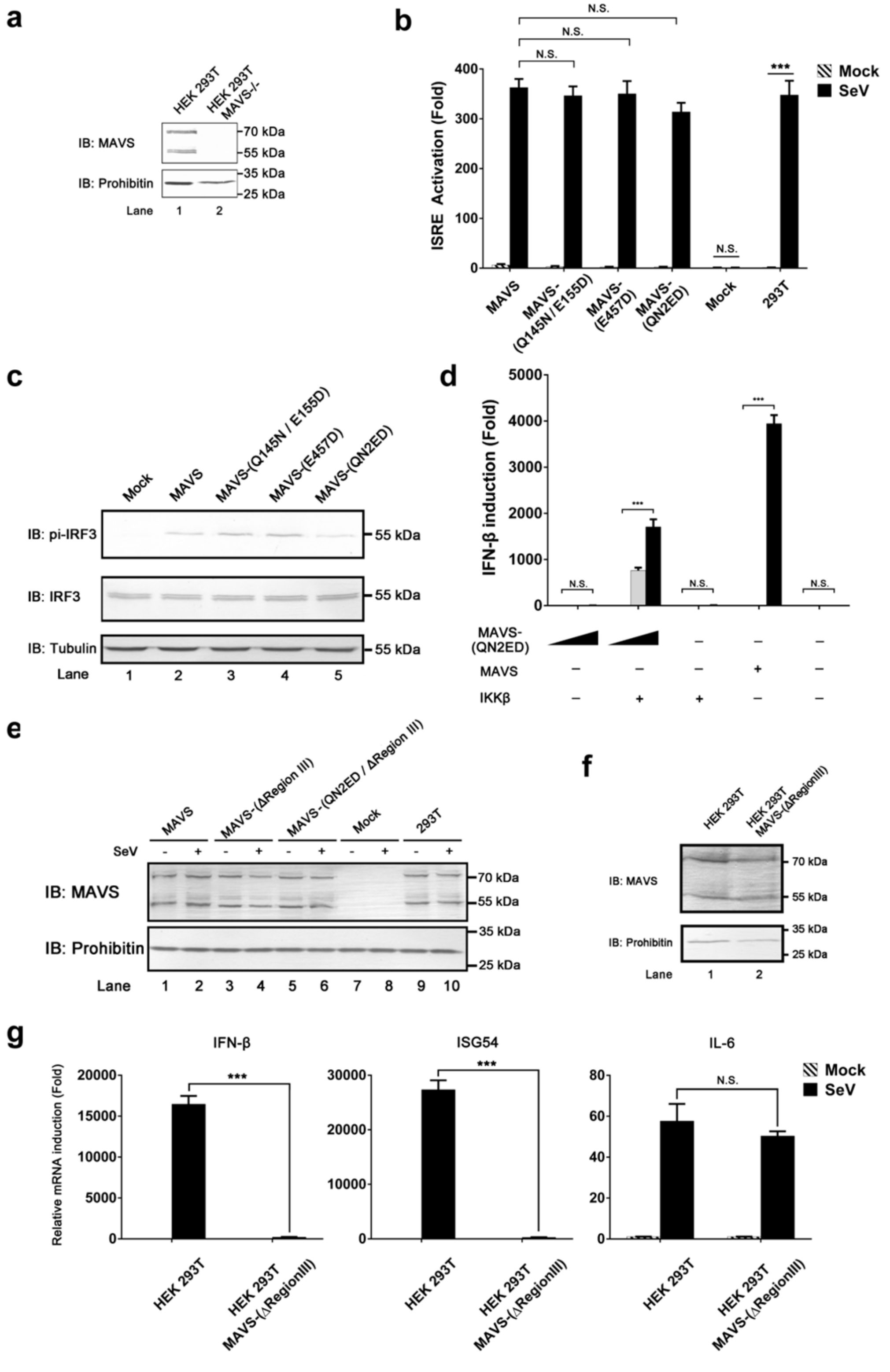

Supplementary figure 1 | MAVS harbors a region with TBK1/IRF3-stimulating activity other than its putative TRAF-binding sites . (**a**) P5 was isolated from HEK 293T or MAVS-deficient HEK 293T cells, which was used for western blot with anti-MAVS and anti-prohibitin antibodies. The original full blot can be found in Supplementary Fig. 6d. (**b**) Samples were made as described in Figure 1(**b**) and firefly luciferase inductions were measured. (**c**) Various MAVS forms were transduced into *MAVS-/-* MEF cells, which were harvested for following analysis thirty-six hours after transduction. IRF3 phosphorylation was examined by western blotting with anti-pi-IRF3 antibody. Total protein level of IRF3 was also examined. The original full blot can be found in Supplementary Fig. 6e. (**d**) MAVS with TRAF-binding motifs disrupted could still stimulate TBK1/IRF3. MAVS-(QN2ED) with or without IKKβ were expressed in *MAVS-/-* HEK 293T cells and IFN expressions were measured by qPCR. (**e**) MAVS expression level was detected for samples as described in Figure 1(**f**) (**g**) (**h**). The original full blot can be found in Supplementary Fig. 7a. (**f**) A cell line (*MAVS-(ΔRegion III)* 293T cells) was made with Region III of endogenous MAVS knocked-out. The original full blot can be found in Supplementary Fig. 7b. (**g**) *MAVS-(ΔRegion III)* 293T cells were infected with or without Sendai virus. Twelve hours after infection, ISG54, IL-6 and IFN induction were measured with qPCR.

Supplementary Figure 2

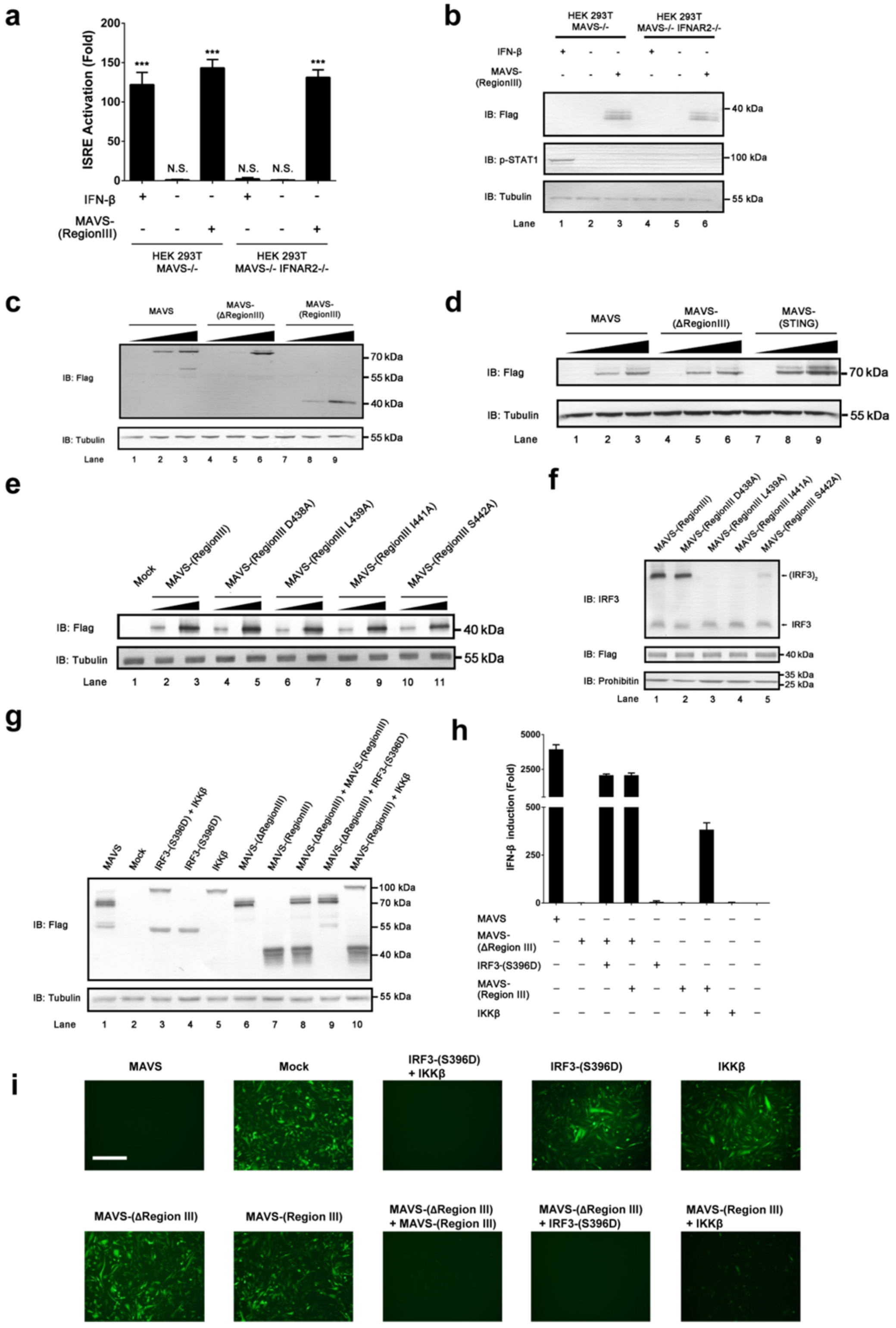

Supplementary figure 2 | Characterization of the TBK1/IRF3-stimulating region of MAVS. (**a**, **b**) ISRE-luciferase reporter, together with or without MAVS-(Region III) were transfected into *MAVS-/-* HEK 293T cells or *IFNAR2-/- & MAVS-/-* HEK 293T cells, which were then treated with or without IFN-β (10 U per ml). The cells were harvested for following analysis twelve hours after treatment. Firefly luciferase inductions were measured (**a**). Protein expression levels were analyzed (**b**). The original full blot can be found in Supplementary Fig. 7c. (**c**) Protein expression levels were shown for samples described in Figure 2b. The original full blot can be found in Supplementary Fig. 7d. (**d**) Protein expression levels were shown for samples described in Figure 2d. The original full blot can be found in Supplementary Fig. 7e. (**e**) Protein expression levels were indicated for samples described in Figure 2e. The original full blot can be found in Supplementary Fig. 7f. (**f**) Various amounts of pcDNA3-flag-MAVS-(Region III) mutants were transfected into *MAVS-/-* HEK 293T cells. Thirty-six hours after transfection, P5 fractions were isolated from the cells and IRF3 dimerization was performed *in vitro*. The original full blot can be found in Supplementary Fig. 8a. (**g**) Protein expression levels were detected for samples described in Figure 2f. The original full blot can be found in Supplementary Fig. 8b. (**h**) Expression vectors as indicated were transfected into *MAVS-/-* HEK 293T cells and IFN expression were measured by qPCR. (**i**) MAVS without Region III is defective in its antiviral function, which could be rescued by coexpression of MAVS (Region III) or a

constitutively active form of IRF3-(S396D). Various MAVS forms were transfected into *MAVS-/-* HEK 293T cells. Thirty-six hours after transfection, cells were infected with VSV-ΔM51-GFP. Fluorescent images were taken eight hours after VSV infection to visualize GFP-positive cells, indicating VSV proliferation. Scale bar represents 500 micrometers.

Supplementary Figure 3

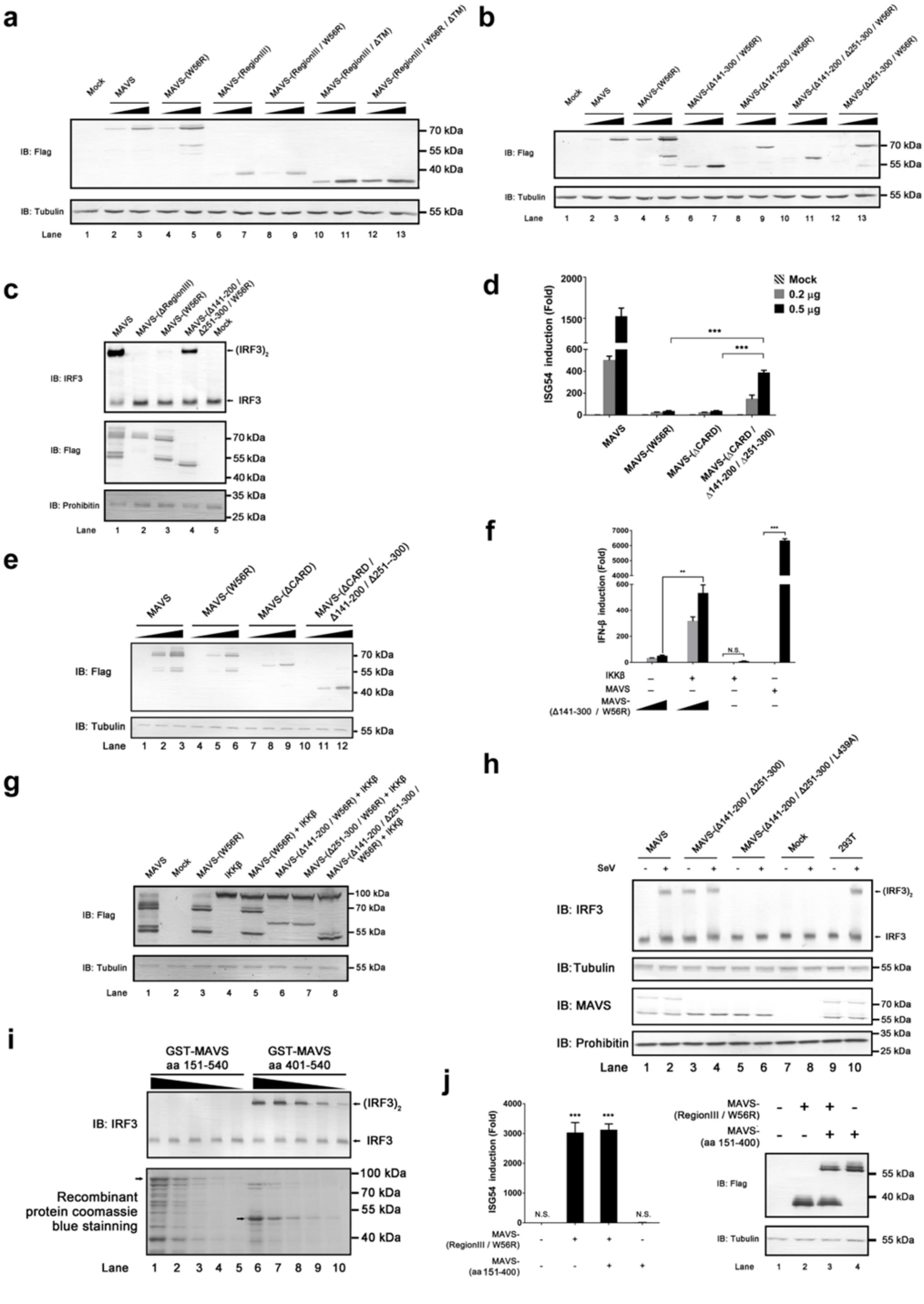

Supplementary figure 3 | The activity of MAVS Region III is inhibited by leading region aa-141-300 in its quiescent state. (**a**) Protein expression levels were detected for samples described in Figure 3b. The original full blot

can be found in Supplementary Fig. 8c. (**b**) Protein expression levels were detected for samples described in Figure 3c. The original full blot can be found in Supplementary Fig. 8d. (**c**) pcDNA3-flag-MAVS (wild type and mutants) were transfected into *MAVS-/-* HEK 293T cells. P5 was isolated thirty-six hours after transfection to perform IRF3 dimerization assay *in vitro*. The original full blot can be found in Supplementary Fig. 8e. (**d**, **e**) Various amounts of constructs as indicated were transfected into *MAVS-/-* HEK 293T cells. Gene inductions were measured thirty-six hours after transfection (**d**). Protein expression levels are shown (**e**). The original full blot can be found in Supplementary Fig. 8f. (**f**) Coexpression of MAVS-(∆aa-141-300 / W56R) and IKKβ induced IFN expression. Various expression vectors as indicated were transfected into *MAVS-/-* HEK 293T cells and IFN inductions were measured with qPCR. (**g**) Protein expression levels were detected for samples described in Figure 3e. The original full blot can be found in Supplementary Fig. 9a. (**h**) Protein expression levels were detected for samples described in Figure 3f. The original full blot can be found in Supplementary Fig. 9b. (**i**) Recombinant proteins GST-MAVS-(aa-151-400) and GST-MAVS-(aa-401-540) were expressed and purified from *E. coli*. Increasing amounts of GST-MAVS-(aa-151-400) or GST-MAVS-(aa-401-540) were used for IRF3 dimerization assay *in vitro*. The original full blot can be found in Supplementary Fig. 9c. (**j**) Constructs as indicated were transfected into *MAVS-/-* HEK 293T cells. Gene inductions were measured thirty-six hours after transfection (left).

Protein expression levels are shown (right). The original full blot can be found in Supplementary Fig. 9d.

Supplementary Figure 4

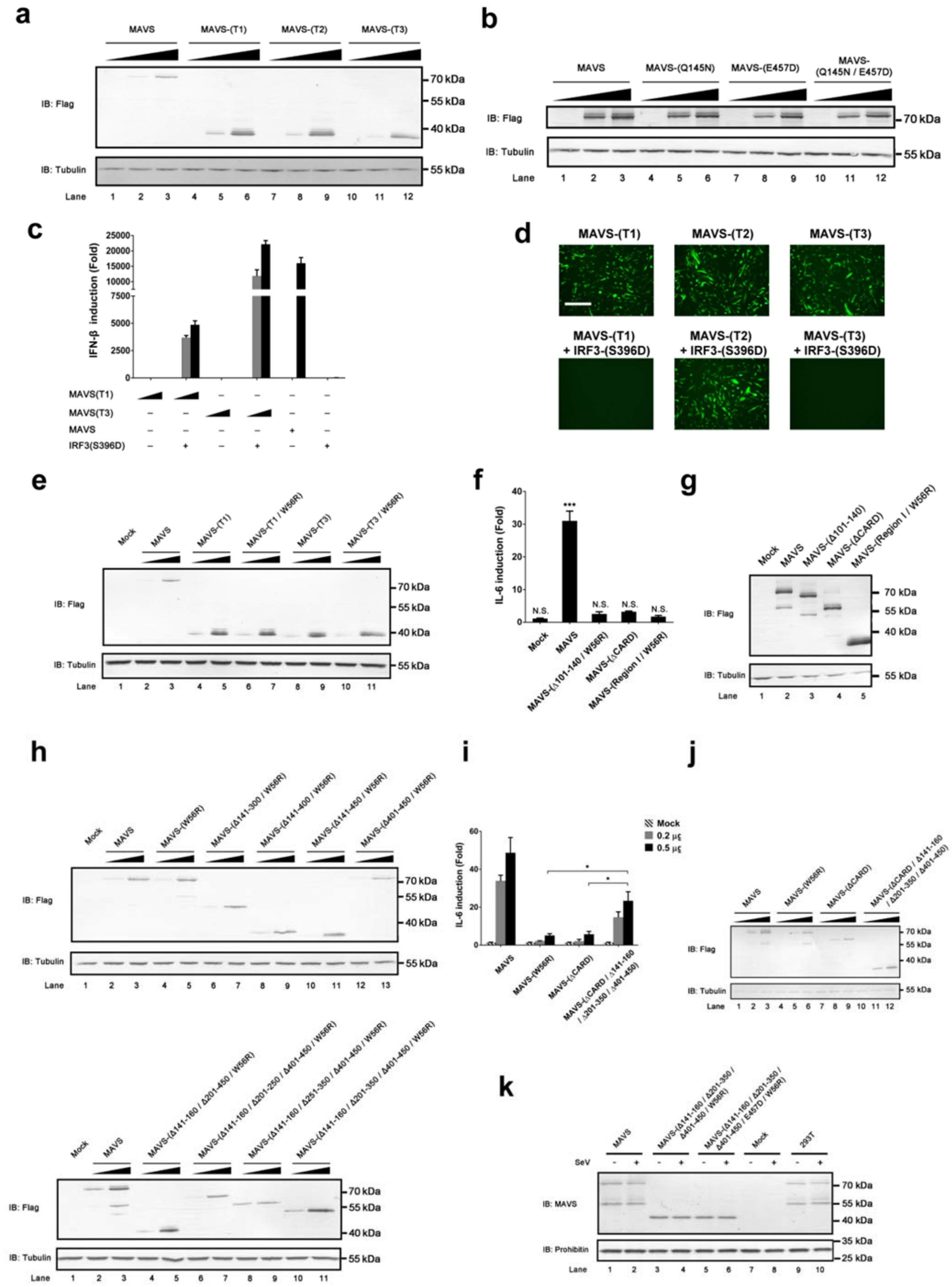

Supplementary figure 4 | Identification of two NF-κB-stimulating regions in MAVS. (**a**) Protein expression levels were detected for samples described in Fig. 4b. The original full blot can be found in Supplementary Fig. 9e. (**b**) Protein expression levels were detected for samples described in Fig.

4c. The original full blot can be found in Supplementary Fig. 9f. (**c**) Coexpression of MAVS (T1) and MAVS (T3) with IRF3 (S396D) induced IFN expression. A combination of plasmids as indicated were transfected into *MAVS-/-* HEK 293T cells and IFN inductions were measured with qPCR thirty-six hours after transfection. (**d**) In the presence of IRF3 (S396D), both MAVS (T1) and MAVS (T3) could provide antiviral activity. Expression vectors as indicated were transduced into *MAVS-/-* MEF cells followed by VSV-ΔM51-GFP infection. Fluorescent images were taken twenty-four hours post virus infection to examine VSV proliferation. Scale bar represents 500 micrometers. (**e**) Protein expression levels were detected for samples described in Fig. 4d. The original full blot can be found in Supplementary Fig. 10a. (**f, g**) Constructs as indicated were transfected into *MAVS-/-* HEK 293T cells. Gene inductions were measured thirty-six hours after transfection (**f**). Protein expression levels are shown (**g**). The original full blot can be found in Supplementary Fig. 10b. (**h**) Protein expression levels were detected for samples described in Fig. 4e. The original full blot can be found in Supplementary Fig. 10c-d. (**i, j**) Various amounts of constructs as indicated were transfected into *MAVS-/-* HEK 293T cells. Gene inductions were measured thirty-six hours after transfection (**i**). Protein expression levels are shown (**j**). The original full blot can be found in Supplementary Fig. 10e. (**k**) Protein expression levels were detected for samples described in Fig. 4f. The original full blot can be found in Supplementary Fig. 10f.

Supplementary Figure 5

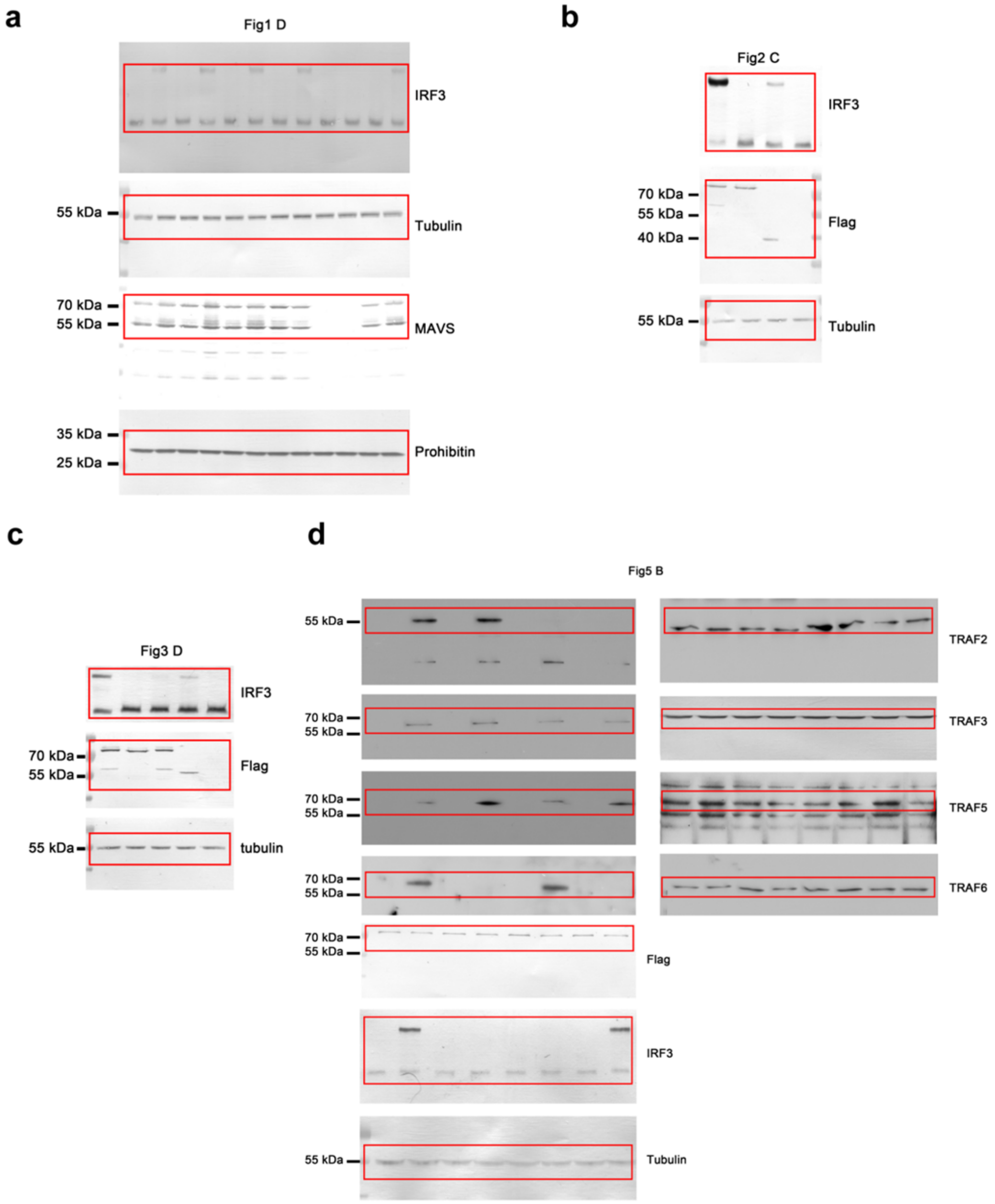

Supplementary figure 5 | Full blot. (**a**) For Fig. 1d. (**b**) For Fig. 2c. (**c**) For Fig. 3d. (**d**) For Fig. 5b.

Supplementary Figure 6

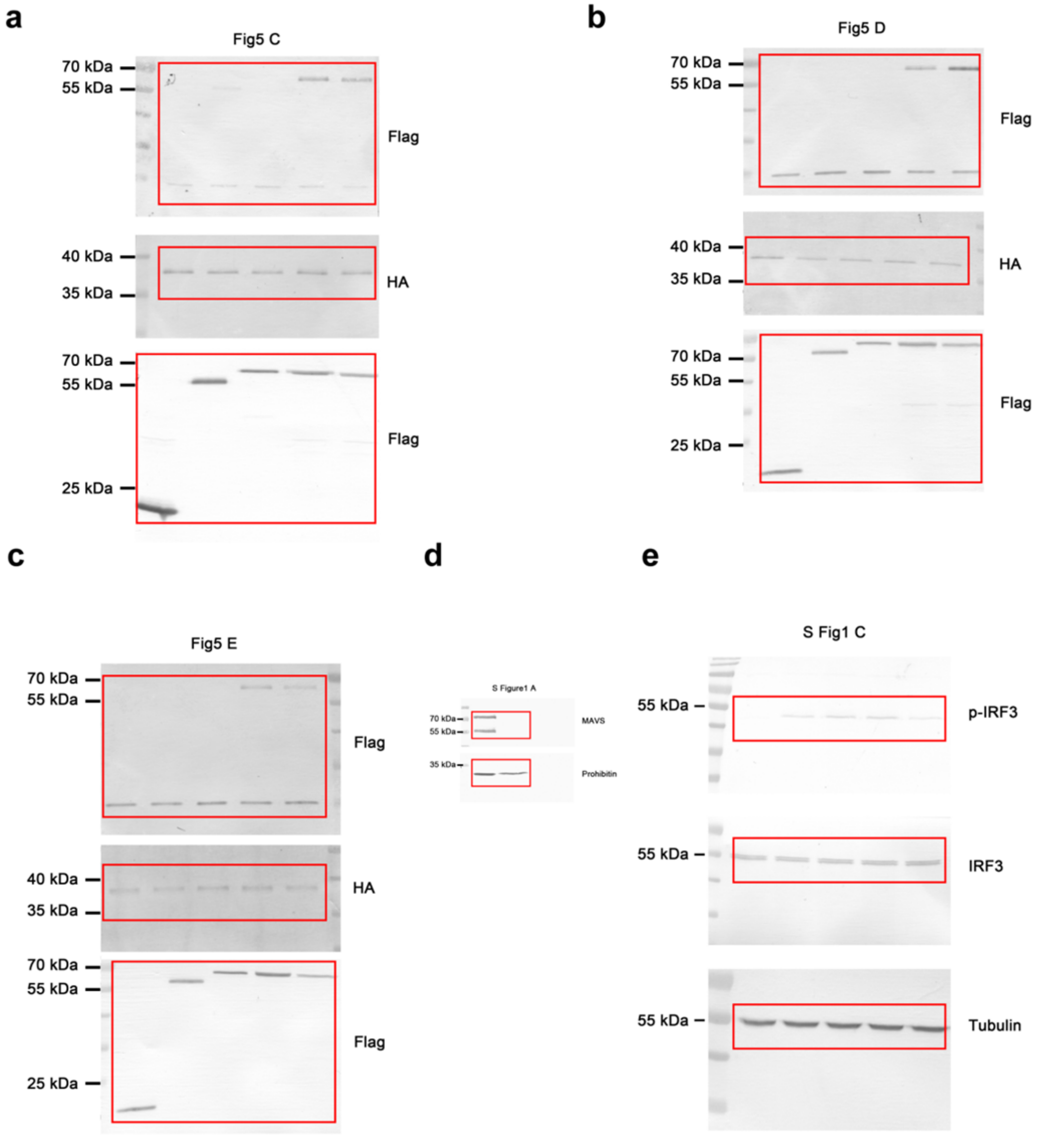

Supplementary figure 6 | Full blot. (**a**) For Fig. 5c. (**b**) For Fig. 5d. (**c**) For Fig. 5e. (**d**) For Supplementary Fig. 1a. (**e**) For Supplementary Fig. 1c.

Supplementary Figure 7

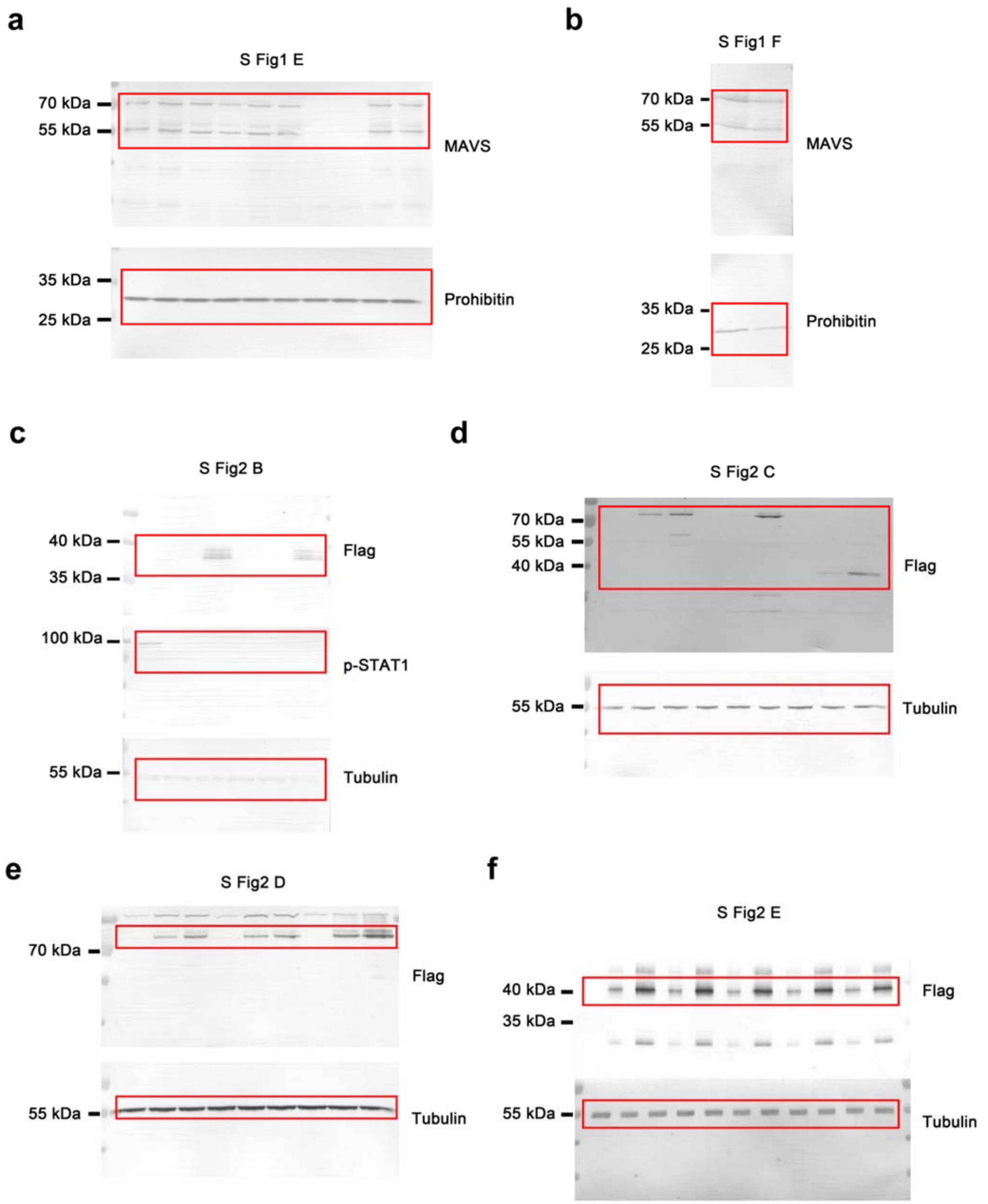

Supplementary figure 7 | Full blot. (**a**) For Supplementary Fig. 1e. (**b**) For Supplementary Fig. 1f. (**c**) For Supplementary Fig. 2b. (**d**) For Supplementary Fig. 2c. (**e**) For Supplementary Fig. 2d. (**f**) For Supplementary Fig. 2e.

Supplementary Figure 8

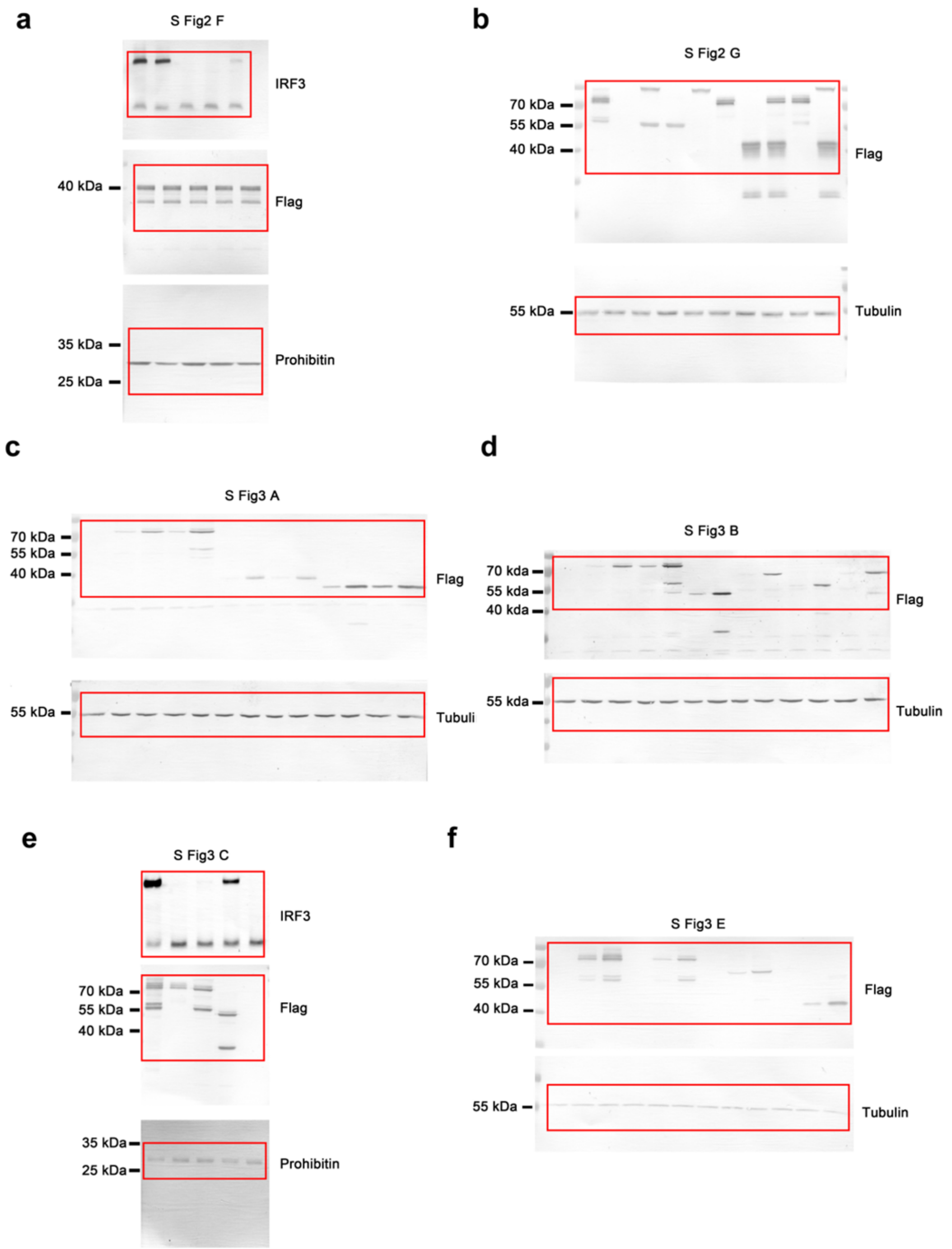

Supplementary figure 8 | Full blot. (**a**) For Supplementary Fig. 2f. (**b**) For Supplementary Fig. 2g. (**c**) For Supplementary Fig. 3a. (**d**) For Supplementary Fig. 3b. (**e**) For Supplementary Fig. 3c. (**f**) For Supplementary Fig. 3e.

Supplementary Figure 9

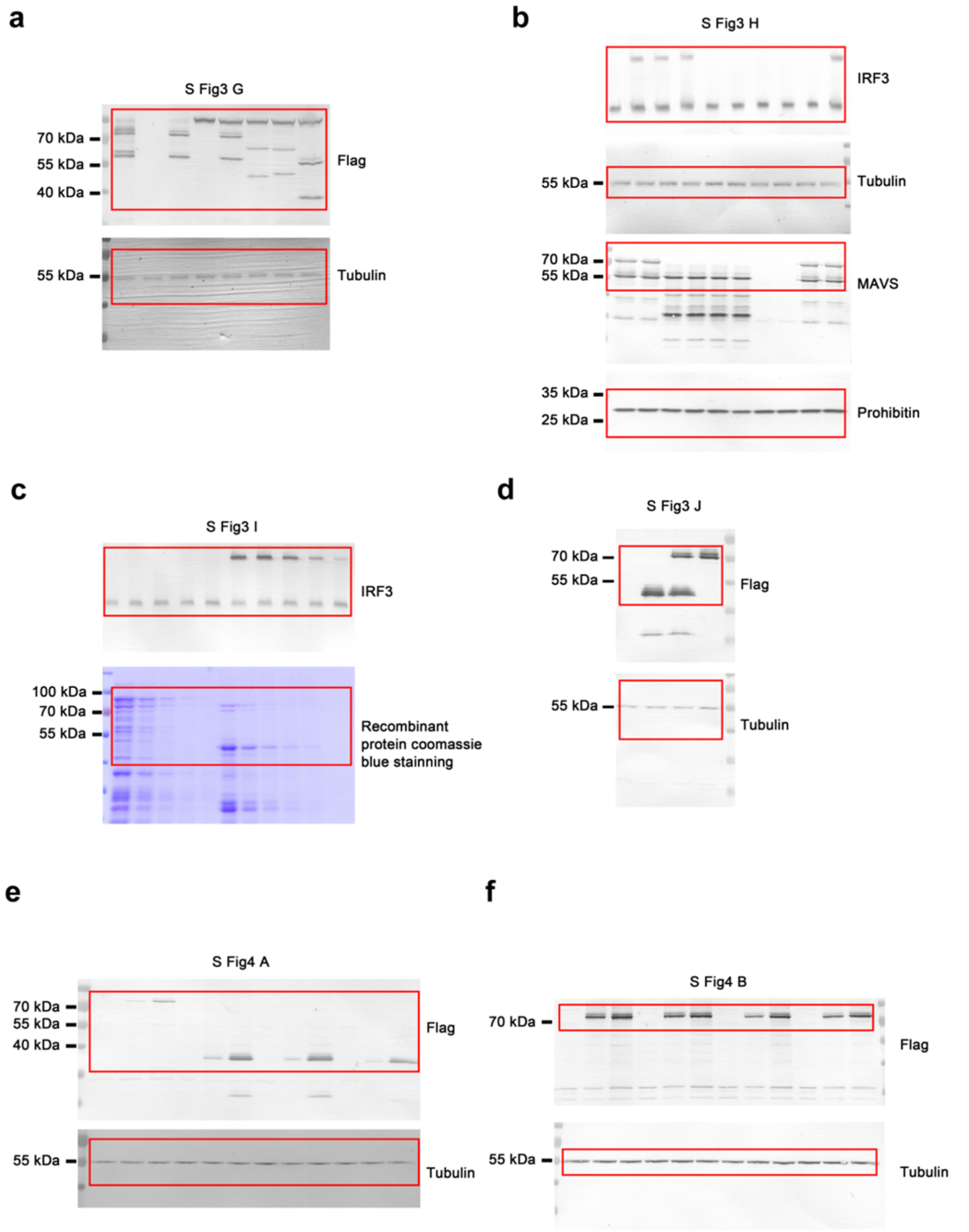

Supplementary figure 9 | Full blot. (**a**) For Supplementary Fig. 3g. (**b**) For Supplementary Fig. 3h. (**c**) For Supplementary Fig. 3i. (**d**) For Supplementary Fig. 3j. (**e**) For Supplementary Fig. 4a. (**f**) For Supplementary Fig. 4b.

Supplementary Figure 10

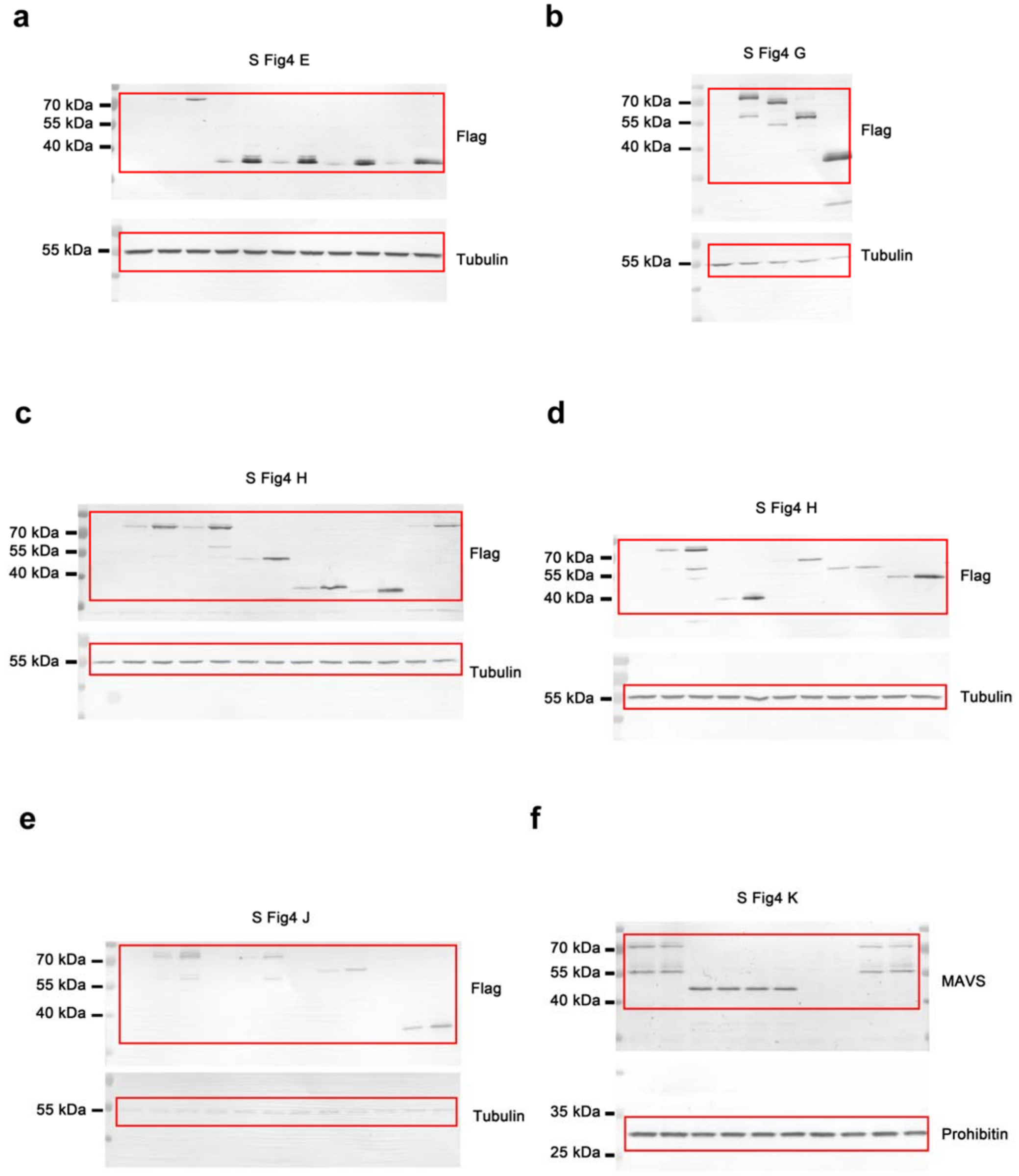

Supplementary figure 10 | Full blot. (**a**) For Supplementary Fig. 4e. (**b**) For Supplementary Fig. 4g. (**c**) For Supplementary Fig. 4h (upper). (**d**) For Supplementary Fig. 4h (lower). (**e**) For Supplementary Fig. 4j. (**f**) For Supplementary Fig. 4k.

Supplementary Table 1│ Primers

Primer Name	Primer sequence (from 5' to 3')
MAVS E457D 5'	gccatggcccagaggataatgagtataagtccgaaggcaccttt
MAVS E457D 3'	atcctctgggccatggcaagg
MAVS Δ 401-450 5'	ccggcgccactggaggaccctgtcatggacctgaggagaatgag
MAVS Δ 401-450 3'	tcctccagtggcgccggcgg
MAVS 401-450 5'	tttggtaccatggactacaaggacgacgatgacaagagttcggcc tggctagacagcag
MAVS 401-450 3'	tttgaattcccccatgcccaaggaggtgc
MAVS W56R 5’	cagggaaccgggacacactcaggcatctcttcaatacccttcagc
MAVS W56R 3’	tgagtgtgtcccggttccctgagagtg
IKK-beta 5’	tacggtaccatggactacaaggacgacgatgataagatgagctg gtcaccttccctg
IKK-beta 3’	atcgtctagactatgaggcttgctccaggc
MAVS Q145N,E155D 5’	aatgaaacccaagcgccagagtcccccggagacaattc
MAVS Q145N,E155D 3’	gtctccgggggactctggcgcttgggtttcattgac
MAVS Δ141-400 5’	gcagagagaaggaaccaagttacagctcagcctggctagacag cag
MAVS Δ141-400 3’	gtaacttggctccttctctctgcagc
MAVS Δ251-300 5’	cccacaggatcagttgtatctactaccttgatgcccgtgaacacagt gg
MAVS Δ251-300 3’	agtagatacaactgatcctgtgggtcc
MAVS Δ101-140 5’	ctaccagcctcggacctcgcccatgcctgtgcaggagacc
MAVS Δ101-140 3’	cgaggtccgaggctggtagctctc
MAVS Δ141-150 5’	cagagagaaggagccaagttacgagtccccaggagagaattca gag
MAVS Δ141-150 3’	gtaacttggctccttctctctgcagc
MAVS Δ141-300 5’	cagagagaaggagccaagttacacgttgatgcctgtgaacacag tgg
MAVS Δ201-300 5’	gggcaccaggaaaaggacactactctcatgcctgtgaacacagt gg
MAVS Δ201-300 3’	agtgtccttttcctggtgcccgctg
MAVS Q145N 5’	caagttaccccatgcctgtcaatgagacccaagcgccagag
MAVS Q145N 3’	attgacaggcatggggtaacttggctc
MAVS Δ141-450 3’	ctctgggccgtggcaggggtaacttggctccttctctctgcag
MAVS Δ201-450 3’	ctctgggccatgacacggtgtgtccttctcctgatgcccgc
MAVS Δ301-450 3’	ctctgggccatggcagggggtagggactttggagggcagag
MAVS Δ251-350 5’	cccacaggatcagttgtatctactatcaattcaacccgtgctggc
MAVS Δ201-250 5’	gggcaccaggaaaaggacacaggcacatcattctcctcctcatc c
MAVS Δ201-350 5’	gggcaccaggaaaaggacactatcaactcaacccgtgctggc
MAVS Δ141-200 5’	cagagagaaggagccaagttacgaactcggaagtacccacac agcag

**MAVS-500 FLAG 5’**	gactacaaggacgacgatgacaagtttcaggagagggaggtgc catgc
**MAVS CARD FLAG 3’**	cttgtcatcgtcgtccttgtagtcagaggtccgaggctggtagctc
**MAVS 500 XhoI 3’**	atactcgagcttccggtctgcttgtggcctgg
**MAVS STING SWAP 3’**	ctctgggccatggcaggggggagaaatccgtgcggagaggg
**MAVS STING SWAP 5’**	gccggcgcaactggaggagttactgtgggcagcttgaagacc
**MAVS 450 5’**	ccctgccatggcccagaggag
**MAVS 400 3’**	tcctccagttgcgccggcgg
**Region III aa437 3’**	ttcaaagcagcccgagaacg
**Region III D438A 5’**	cgttctcgggctgctttgaagctcttgccatcagcgccagcac
**Region III L439A 5’**	cgttctcgggctgctttgaagacgctgccatcagtgctagcacctcc
**Region III I441A 5’**	cgttctcgggctgctttgaagatcttgcggcaagtgctagcacctcct tgggc
**Region III S442A 5’**	cgttctcgggctgctttgaagatcttgccatcgctgctagcacctcctt gggcatgg
**Region III S442Y 5’**	cgttctcgggctgctttgaagatcttgccatctacgccagcacctcctt gggcatgg
**MAVS aa-151-540 5’**	ttaggatccgagtccccaggagagaattcagag
**MAVS aa-401-540 5’**	tttggatccagctcagcatggctagacagcag
**MAVS aa-540 3’**	tttctcgagctagtgcagacgccgccggtac
**TRAF2 5'**	ctgaggtaccatggcagcagctagcgtgac
**TRAF2 3'**	acgatctagattaaagcccagtcaggtccacaatg
**TRAF3 5'**	tgaggtaccatggagtcgagtaaaaagatgga
**TRAF3 3'**	tacactcgagtcaggggtcaggcagatccg
**TRAF5 5'**	gacactcgagatggcttactcagaagaacataaagg
**TRAF5 3'**	agcgtctagactagagatcctccaggtcagttaag
**TRAF6 5'**	ctgaggtaccatgagtctcctaaactgtgaaaacagctg
**TRAF6 3'**	tacactcgagctataccccctgcatcagtacttcgtgg
**IRF3 S396D 5'**	tgcatattgacaacagccacccactcc
**IRF3 S396D 3'**	ggtggctgtcaatatgcaggtccacagtatt
